# Infection rate among nutritional therapies for acute pancreatitis: A systematic review with network meta-analysis of randomized controlled trials

**DOI:** 10.1371/journal.pone.0219151

**Published:** 2019-07-10

**Authors:** Ping-Han Hsieh, Hsiu-Yueh Su, Chung-Yuan Lin, Yi-No Kang, Chun-Chao Chang

**Affiliations:** 1 Department of Medicine, Taipei Veterans General Hospital, Taipei, Republic of China (Taiwan); 2 Center for Evidence-Based Medicine, Department of Education, Taipei Medical University Hospital, Taipei, Republic of China (Taiwan); 3 Department of Dietetics, Taipei Medical University Hospital, Taipei, Republic of China (Taiwan); 4 School of Medicine, College of Medicine, Taipei Medical University, Taipei, Republic of China (Taiwan); 5 Center, Wan-Fang Hospital, Taipei Medical University, Taipei, Republic of China (Taiwan); 6 Division of Gastroenterology and Hepatology, Department of Internal Medicine, Taipei Medical University Hospital, Taipei, Republic of China (Taiwan); 7 Division of Gastroenterology and Hepatology, Department of Internal Medicine, School of Medicine, Taipei Medical University, Taipei, Republic of China (Taiwan); University of Mississippi Medical Center, UNITED STATES

## Abstract

**Background:**

Infection in acute pancreatitis (AP) is associated with nutritional therapies including naso-gastric (NG), naso-jejunal (NJ), and total parenteral nutrition (TPN). To examine infections among NG, NJ, TPN, and no nutritional support (NNS) in treating patients with AP.

**Methods:**

The investigators completed comprehensive search in the Cochrane library, EMBASE, PubMed, Web of Science, and ClinicalTrials.gov without restriction on language and publication date before January 21, 2019. They also searched the reference lists of relevant studies for randomized controlled trials (RCTs) comparing NG, NJ, TPN, and NNS among patients with AP. Quantitative synthesis was conducted in a contrast-based network meta-analysis. To clarify effects, a network meta-analysis was conducted to calculate the surface under the cumulative ranking curve (SUCRA). Beside of overall infections, the event rates of infected pancreatic necrosis, bacteremia, line infection, pneumonia, urinary tract infection, and other types of infections were measured.

**Results:**

The network meta-analysis of 16 RCTs showed that NJ had significantly lower overall infection rates compared with TPN (risk ratio: 0.59; 95% confidence interval: 0.38, 0.90); and NG had a larger effect size and higher rank probability compared with NJ, TPN, and NNS (mean rank = 1.7; SUCRA = 75.8). TPN was the least preferred (mean rank = 3.2; SUCRA = 26.6).

**Conclusions:**

NG and NJ may be preferred therapies for treating patients with AP. Clinicians may consider NG as a first-line treatment for patients with AP (including severe AP) and even in patients receiving prophylactic antibiotics. In addition, we found that NNS should be avoided when treating patients with severe AP.

## Introduction

Nutritional therapy is an important topic for patients with acute pancreatitis, and it covers standard therapy and nutritional therapy.[1] Standard therapy refers to when patients can tolerate it. Nutritional therapy involves three routes including nasogastric tube (NG) feeding, nasojejunal tube (NJ) feeding enteral and parenteral nutrition. NG feeding and NJ feeding are enteral nutrition. Parenteral nutrition can be provided independently (total parenteral nutrition, TPN), or be combined with enteral nutrition. Numerous randomized controlled trials (RCTs) and systematic review have attempted to determine the most effective nutrition therapy among NG, NJ, TPN, and NNS in past decades.[2, 3] Although the guideline suggested that enteral nutrition is superior to TPN in terms of reducing the infectious complication of predicted severe acute pancreatitis (pSAP),[4] further discussion is still required.

Firstly, recommendations about nutritional routes for acute pancreatitis usually are based on relevant systematic reviews.[2, 3, 5] They included important RCTs.[6–13] Nevertheless, the previous systematic reviews of enteral nutrition typically refer to the nasojejunal route. As we know, NG is located differently from NJ and may directly stimulate pancreatic exocrine secretion or even exacerbate pancreatic inflammation.[14] Thus, the definitions of enteral routes require clarification, and NJ and NG feeding should be discussed separately. Secondly, two systematic reviews cited by the guideline performed several errors.[4, 15] Data in the two systematic reviews differed from the original reports. For instance, we found that the data for local septic complications (infected necrosis and pancreatic abscess formation) in the TPN differs from the original report by Petrov et al.,[2, 5, 12] and data for infectious complication also differ from the article by Eckerwall et al. [3, 8] The errors should be corrected. Thirdly, there is a need for understanding the effects of prophylactic antibiotics for reducing infections in patients with pSAP,[16, 17] but previous systematic reviews did not considered prophylactic antibiotics usage in their analyses. Moreover, no evidence synthesized NG, NJ, TPN, and NNS in consistency model.

Therefore, we aimed to determine the safety of various nutritional therapies for acute pancreatitis through network meta-analysis of infectious complications. In addition, we also explored the influence from severity and prophylactic antibiotics usage. Our results may provide a clear and practical guidance to nutritional therapies for treating patients with acute pancreatitis.

## Methods

This systematic review with network meta-analysis was conducted by a multi-disciplinary research team involving gastroenterologist, dietitian, and an experienced researcher in systematic review and network meta-analysis.[18, 19] The experienced researcher also participated in gastroenterological studies.[20, 21] Our team followed the PRISMA guidelines, and the study protocol was registered on PROSPERO (CRD42017084125): https://www.crd.york.ac.uk/prospero/display_record.php?RecordID=84125.

### Data sources and searches

We selected RCTs of nutritional therapies for acute pancreatitis from the EMBASE, PubMed, Web of Science, and Cochrane library databases. The primary systematic search strategy with relevant terms was completed in PubMed and was adapted to other databases before January 21st, 2019 (S1 Table). The search strategy consisted of free-text, medical subject headings (MeSH in PubMed and Emtree in EMBASE), abbreviations, appropriate Boolean algebras, and no restrictions on language and publication date.

### Study selection

Two authors (P.H.H and Y.N.K) screened citations identified by systematic searches. Disagreement during the study selection was resolved through discussion. Inclusion criteria for the selection were defined beforehand as follows: (1) patients with pancreatitis; (2) treated by NNS, TPN, or enteral therapies; and (3) RCT. This study used kappa coefficient for thee inter-rater reliability for study selection. The discussion for disagreement involved four steps including (1) reading methods together, (2) explaining why the author would like to include, (3) explaining why the other author would like to exclude, and (4) making decision together.

### Data extraction and quality assessment

Two authors (P.H.H and C.Y.L) individually reviewed the included RCTs for quality evaluation and data extraction. The quality of the RCTs was evaluated using the Cochrane risk of bias tool. Disagreements on the risk of bias evaluations were resolved through discussion. The authors identified relevant information and extracted outcome data. The relevant information included age, prophylactic antibiotics, and severity of acute pancreatitis. Data on infectious events from previous meta-analyses did not consider differences between the numbers of infectious patients and infectious events. Unclear definitions may lead to unclear or mistaken outcomes; therefore, we used the clear definition of the number of infectious patients as a standard counting unit in all analyses. The outcomes of this systematic review were overall infections (total number of infectious patients), infected pancreatic necrosis, bacteremia, line infection, pneumonia, urinary tract infections, and other types of infection (OTIs). The OTIs included bile culture, sepsis, unspecified drain, and wound infection.

### Data synthesis and analysis

We performed a quantitative synthesis through a meta-analysis, which used the risk ratio (RR) for binary data and was conducted in a random-effects model. The effect size was calculated using a 95% confidence interval (CI) and *P* value. A *P* value of <0.05 was considered statistically significant in all analyses. A small study bias in the meta-analysis was examined using a funnel plot with Egger’s regression intercept. Inconsistency in the network meta-analysis was examined using the Lu–Ades loop inconsistency test. Network meta-analysis was conducted in STATA version 14.

To examine effects, we calculated the surface under the cumulative ranking curve (SUCRA) and further analyzed the overall infection rate based on the severity of acute pancreatitis and prophylactic antibiotics usage. The SUCRA is a statistical technique with advantage as more than two comparators in a meta-analysis model by demonstrating a hierarchy of intervention rankings. It provides the probability of an intervention being among the most effective interventions.[22] For further analysis of the severity of acute pancreatitis, the network meta-analysis synthesized RCTs that included only patients with pSAP. For further analysis of prophylactic antibiotics, the network meta-analysis synthesized only RCTs that administered prophylactic antibiotics to every patient.

## Results

**Fig 1:**
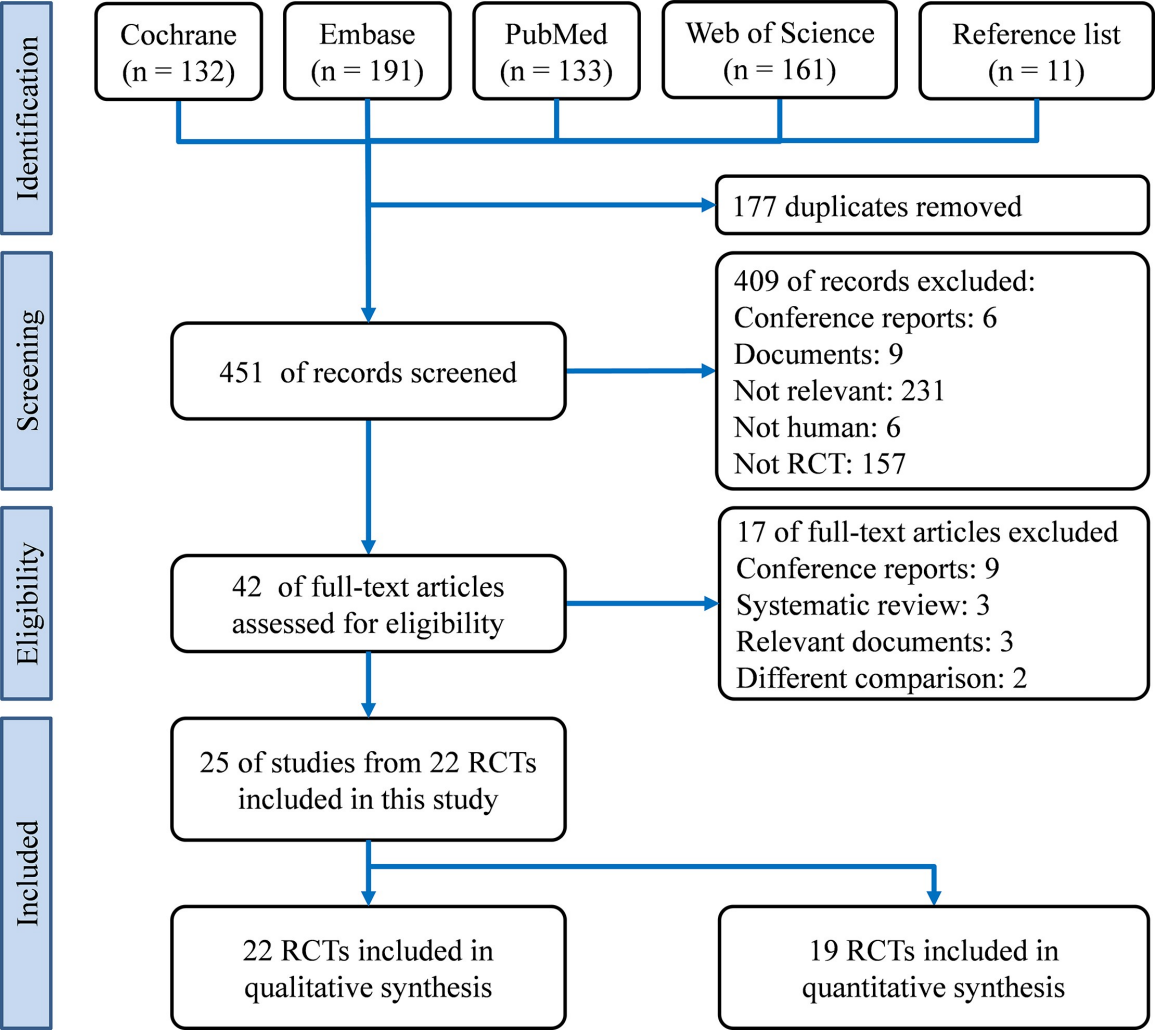
Preferred reporting items for systematic reviews and meta-analyses (PRISMA) flow diagram of study identification process.

A total of 628 potential references were identified in databases through systematic searches (n = 617) and hand search (n = 11), of which 177 were duplicated. Of the remaining 451 references, we excluded 409 after title and abstract screening. Subsequently, we retrieved the full text of 42 references for further review. Finally, we excluded 17 of the 42 references because they met the exclusion criteria. The excluded 17 references were conference abstract without complete information (n = 9),[23–31] systematic review (n = 3),[32–34] relevant document (n = 2),[35–37] and no comparison for nutritional routes (n = 2).[38, 39] Among the 42 references, two authors had agreements on 40 references (25 were included and 15 were excluded), and there were two references were disagreed in the individual review.[38, 39] After the discussion, the two references were excluded because of no comparison between nutritional routes. The kappa coefficient (0.899) reflected that the authors had similar judgement. The 25 eligible references after the full-text review were from 22 RCTs, and they were included in this study for qualitative and quantitative synthesis.[6–13, 40–56] Fig 1 presents a flow diagram of study selection according to PRISMA guidelines.

### Characteristics and quality of included studies

The 22 RCTs included in the study consisted of 1379 patients and were conducted in the United States,[40, 45, 51] Canada,[11] China,[13, 41, 54–56] Croatia,[53] Greece,[10] Hungary,[47] India,[7, 43, 52] New Zealand,[44, 46, 48, 49] Russia,[12] Scotland,[42, 50] Spain,[6] Sweden,[8] and the United Kingdom[9] from 1984 to 2012. These RCTs included 4 therapies: NNS (n = 190), TPN (n = 420), NG (n = 163), and NJ (n = 562). The overall quality of the RCTs is shown in S2 Table. The available information showed that the age of patients in each RCT ranged from 36–72 years. In total, 780 men (58.43%) were included in the RCTs. Eleven RCTs provided all patients with prophylactic antibiotics,[7, 9, 10, 12, 13, 41, 50, 53–56] and 17 RCTs only included patients with SAP (Table 1).[6–13, 41–43, 50, 52–56] Among the 22 trials, two authors had agreements on 264 main extractions (196 information were extracted and 68 items were no information), and there were 22 disagreements in the individual review. The kappa coefficient (0.808) indicated that the agreement between authors was acceptable. Further information is shown in S3 Table.

**Table 1:** Characteristics of the included randomized controlled trials.

		Inclusion	All	All	Sample size				Age				Implementing TPN
Study	Region	period	SAP^1^	PAB	TPN	NNS	NJ	NG	TPN	NNS	NJ	NG	timing
Abou-Assi [40]	USA	2000/1-2000/12			27		26		50 ^2^		48		About 48 hours
Casas [6]	Spain	N/A	✓		11		11		55.6		61.2		Immediate
Doley [7]	India	2006/7-2007/12	✓	✓	25		25		41.1		38.4		Within 72 hours
Du [41]	China	2009/3-2013/12	✓	✓			40	40			43 ^3^	41	N/A
Eckerwall [8]	Sweden	2002/1-2004/12	✓		26			24	68 ^3^			71 ^3^	Within 24 hours
Entock [42]	Scotland	1997/10-2000/7	✓				22	27			58	63 ^3^	N/A
Gupta [9]	UK	1996/11-1998/4	✓	✓	10		11		57		65		About 24 hours
He [55]	China	N/A	✓	✓	22	25			40.2	39.6			Within 48 hours
Kalfarentzos [10]	Greece	1990/7-1995/12	✓	✓	20		18		67.2		63		Within 48 hours
Kumar [43]	India	2002/9-2003/12	✓				14	16			35.57	43.25	N/A
McClave [45]	USA	N/A			16		16		45.1		47.64		Within 48 hours
Louie [11]	Canada	1999/7-2001/12	✓		18		10		59		65.3		Within 24 hours
MIMOSA trial [44, 46, 48, 49]	New Zealand	2010/5-2011/4				18		17		55		41	N/A
Olah [47]	Hungary	1995/1-1996/5			48		41		43.8		47.2		Within 24 hours
Petrov [12]	Russia	2002/3-2004/12	✓	✓	35		35		52 ^3^		51 ^3^		Within 72 hours
Powell [50]	Scotland	1996/12-1998/6	✓	✓		14	13			52	64		N/A
Sax [51]	America	1984/7-1985/12			28	26			39.8	39.8			Within 24 hours
Singh [52]	India	2005/1-2007/12	✓				39	39			39.7	39.1	N/A
Stimac [53]	Croatia	2007/5-2012/2	✓	✓		107	107			72	69 ^3^		N/A
Wang [54]	China	2006/1–2011/12	✓	✓	60		61		41.7		43.15		Within 48 hours
Wu [13]	China	2003/11-2007/12	✓	✓	54		53		54		52		About 48 hours
Zhang [56]	China	2006/1-2009/10	✓	✓	42		42		48.6		47.4		About 72 hours

^1^ N/A, not applicable; NG, naso-gastric; NJ, naso-jejeunal; NNS, no nutrition support; PAB, prophylactic anti-biotics; SAP, severe acute pancreatitis; TPN, total parenteral nutrition.

^2^ Mean (all such values unless otherwise indicated).

^3^ Median.

### Primary outcome: Total number of infectious patients

**Fig 2:**
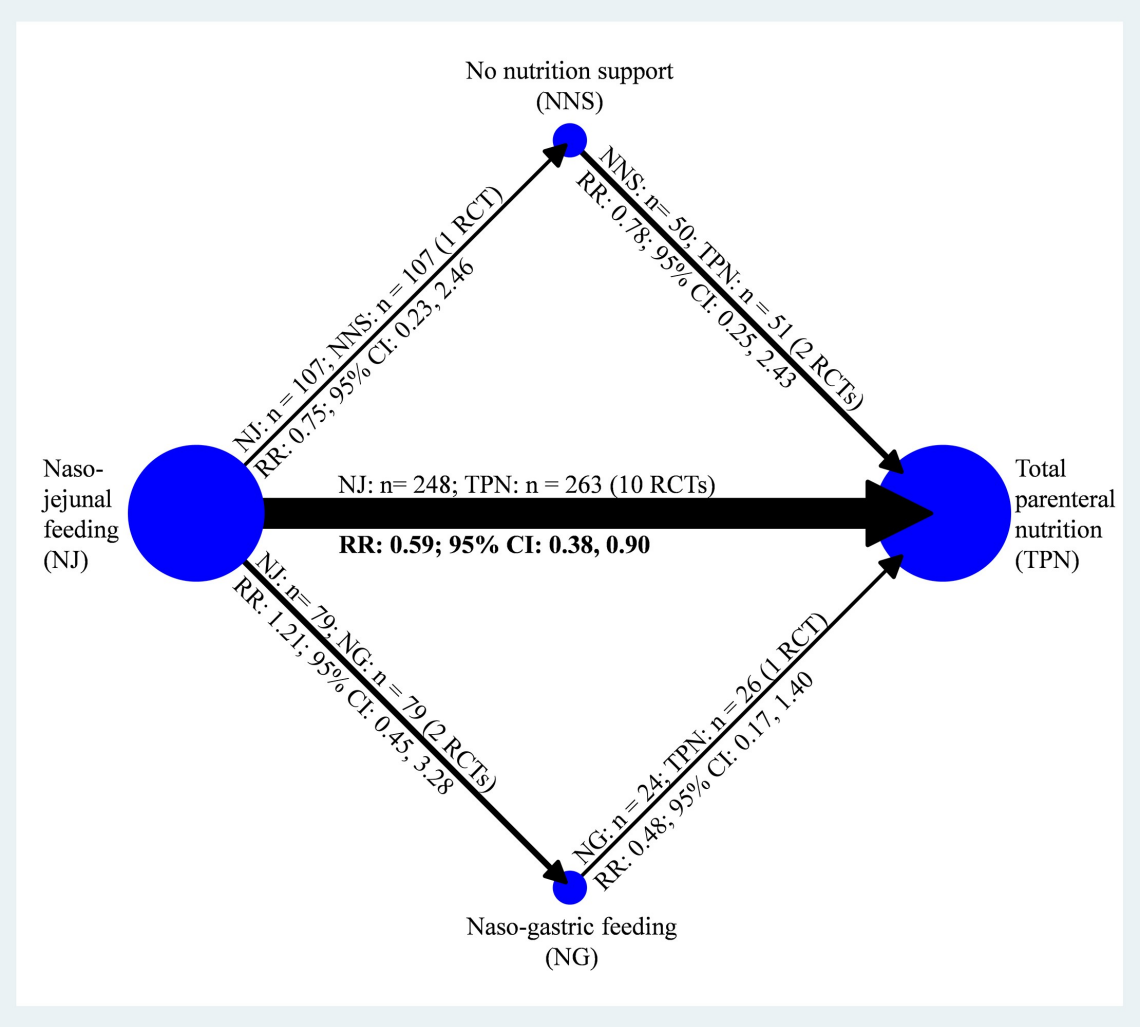
Network geometry of total number of infectious patients among nutritional therapies and no nutritional support.

**Fig 3:**
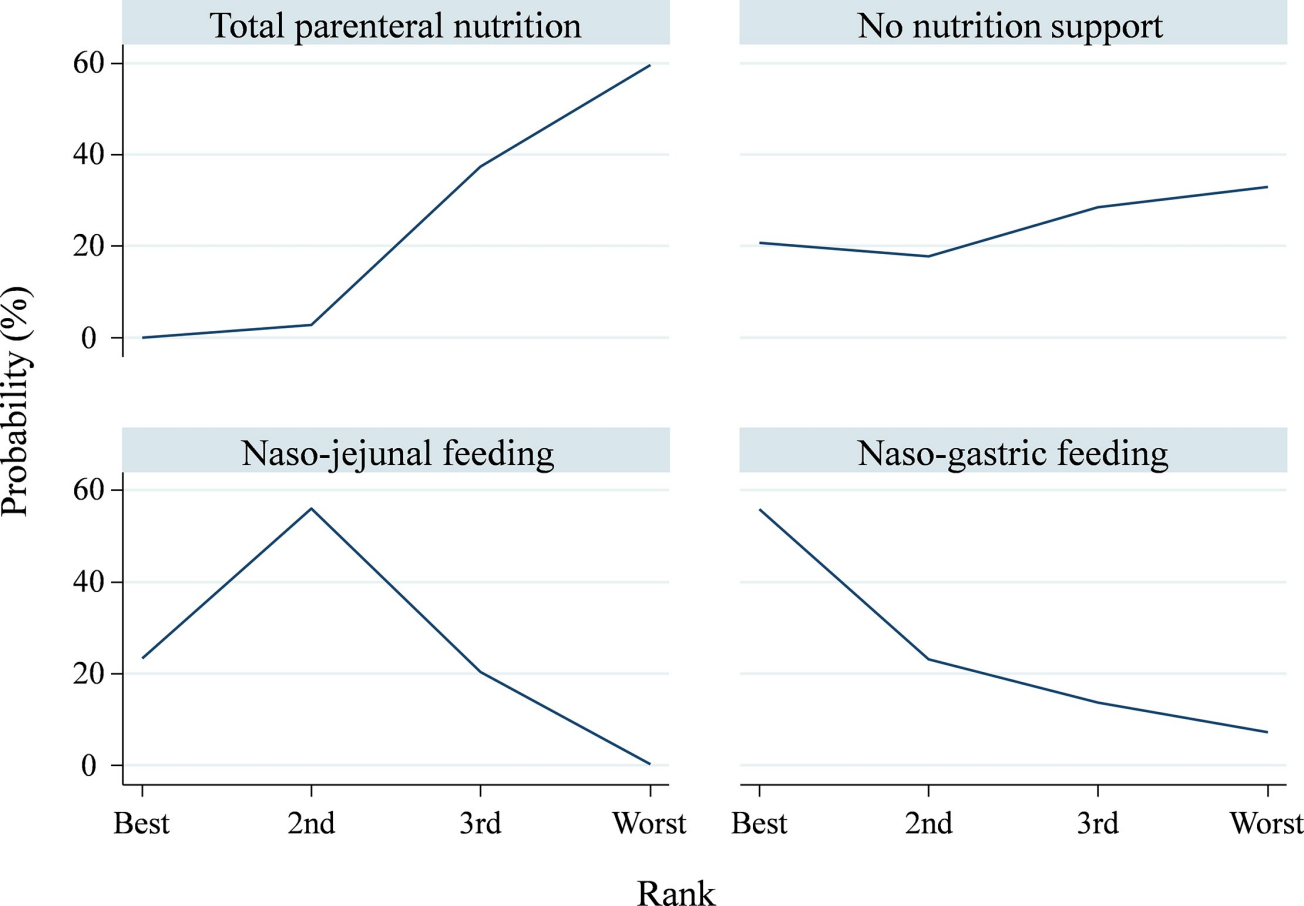
Surface under the cumulative ranking curve of total number of infectious patients among nutritional therapies and no nutritional support.

Of the 22 RCTs, 16 showed data on the total number of infectious patients.[6–12, 41, 45, 47, 51–56] In overall, per infected patient proximately had one to two kind of infections. In the consistency model, the results showed that NJ led to a significantly lower total infection rate compared with TPN (RR, 0.59; 95% CI, 0.38, 0.90), but NG had the largest effect size compared with other therapies (Fig 2 and Appendix A1 in S1 File). The SUCRA value based on cumulative ranking probabilities showed that NG may be the optimal therapy (mean rank = 1.7; SUCRA = 75.8), and TPN should be avoided when treating patients with acute pancreatitis (mean rank = 3.6; SUCRA = 14.5) (Fig 3). To assess influence of inclusion year and TPN implementing timing on the pooled result, meta-regression in network meta-analysis model did not find significant influence (S4 Table). No inconsistency (loop inconsistency *χ*^2^ = 5.40; *P =* 0.07) (Appendix A2 in S1 File) or small study bias (Egger’s test *t* = −1.68; *P =* 0.12) was detected in this consistency model (Appendix A3 in S1 File)

Table 2 presents a summary of further network meta-analysis for total infection (pSAP and prophylactic antibiotics). The 12 RCTs that recruited patients with pSAP showed that the result in the consistency model was similar to the overall pooling.[6–12, 41, 52, 54–56] The NJ led to a significantly lower total infection rate compared with TPN (RR, 0.56; 95% CI, 0.34, 0.92), but NG had the largest effect size compared with other therapies (Table 2 and Appendix B1 in S1 File). Moreover, the SUCRA value showed that NG may be the optimal nutritional route (mean rank = 1.6; SUCRA = 80.9), and TPN should be avoided when treating patients with acute pancreatitis (mean rank = 3.2; SUCRA = 26.6) (Appendix B2 in S1 File). No evidence indicated inconsistency (loop inconsistency *χ*^2^ = 3.68; *P =* 0.06) (Appendix B3 in S1 File) or small study bias (Egger’s test *t* = -1.75; *P =* 0.11) in this network meta-analysis (Appendix B4 in S1 File).

**Table 2:** Summary of further network meta-analysis for total number of infectious patients.

Therapy			Effect size			Inconsistency		Small study bias	
1	2	studies	RR^1^	95% CI	*I*^2^	*χ*^2^	*P*	*t*	*P*
**Total number of infectious patients (pSAP)**						3.68	0.06	-1.75	0.11
NNS	TPN	1	1.41	0.38, 5.16	N/A				
NJ	TPN	8	0.56^2^	0.34, 0.92	45.8%				
NG	TPN	1	0.47	0.16, 1.43	N/A				
NJ	NNS	AIC	0.40	0.10, 1.60	N/A				
NG	NNS	AIC	0.34	0.06, 1.85	N/A				
NG	NJ	2	0.84	0.30, 2.36	0%				
**Total number of infectious patients (PAB)**						2.07	0.15	-1.02	0.34
NNS	TPN	1	0.97	0.28, 3.40	N/A				
NJ	TPN	6	0.59	0.35, 1.00	56.6%				
NG	TPN	AIC	0.19	0.01, 5.30	N/A				
NJ	NNS	1	0.61	0.16, 2.29	N/A				
NG	NNS	AIC	0.19	0.01, 6.16	N/A				
NG	NJ	1	0.32	0.01, 8.61	N/A				
**Infected pancreatic necrosis**						5.70	0.06	0.35	0.73
NNS	TPN	1	1.08	0.43, 2.70	N/A				
NJ	TPN	6	0.39^2^	0.26, 0.58	0%				
NG	TPN	1	0.31^2^	0.11, 0.91	N/A				
NJ	NNS	1	0.36^2^	0.14, 0.97	N/A				
NG	NNS	AIC	0.29	0.07, 1.17	N/A				
NG	NJ	2	0.80	0.29, 2.19	0%				
**Bacteremia**						No	loop	0.51	0.631
NNS	TPN	1	0.15	0.01, 2.83	N/A				
NJ	TPN	4	0.54	0.24, 1.21	0%				
NG	TPN	AIC	0.52	0.17, 1.56	N/A				
NJ	NNS	AIC	3.51	0.17, 72.44	N/A				
NG	NNS	AIC	3.39	0.15, 76.54	N/A				
NG	NJ	2	0.96	0.46, 2.04	0%				
**Line infection**						No	loop	1.15	0.29
NNS	TPN	2	0.79	0.10, 6.38	59.9%				
NJ	TPN	8	0.19^2^	0.07, 0.50	0%				
NJ	NNS	AIC	0.24	0.02, 2.38	N/A				
**Pneumonia**						No	loop	-1.49	0.20
NJ	TPN	4	0.72	0.27, 1.87	0%				
NG	TPN	AIC	0.32	0.07, 1.40	N/A				
NG	NJ	3	0.44	0.14, 1.37	0%				
**Other type infection**						1.83	0.18	-1.52	0.17
NJ	TPN	6	0.87	0.56, 1.34	36.5%				
NG	TPN	1	0.98	0.16, 5.87	N/A				
NG	NJ	2	1.14	0.19, 6.66	53.1%				

^1^ AIC, adjusted indirect comparison; N/A, not applicable; NG, naso-gastric; NJ, naso-jejeunal; NNS, no nutrition support; PAB, prophylactic anti-biotics; pSAP, predicted severe acute pancreatitis; RR, risk ratio; CI, confidence interval; TPN, total parenteral nutrition.

^2^ *P* < 0.05.

In the 9 RCTs that provided all patients prophylactic antibiotics,[7, 9, 10, 12, 41, 53–56] the results of the network meta-analysis showed no significant differences in total infection rates among different therapies (Table 2 and Appendix C1 in S1 File). However, the SUCRA value showed that NG may be the best therapy (mean rank = 1.6; SUCRA = 80.2), and TPN should be avoided when treating patients with acute pancreatitis (mean rank = 3.3; SUCRA = 22.3) (Appendix C2 in S1 File). No inconsistency (loop inconsistency *χ*^2^ = 2.07; *P =* 0.15) (Appendix C3 in S1 File) or small study bias (Egger’s test *t* = -1.02; *P =* 0.34) was observed in the present network meta-analysis (Appendix C4 in S1 File).

### Secondary outcomes

Table 2 summarizes the network meta-analyses for infected pancreatic necrosis, bacteremia, line infection, pneumonia, and OTIs, and their ranking probabilities were summarized in S5 Table. infected pancreatic necrosis was reported in 11 RCTs.[6, 8, 10–13, 43, 52, 53, 55, 56] The network meta-analysis consistently showed that NJ led to a significantly lower infected pancreatic necrosis rate when compared with NNS (RR, 0.36; 95% CI, 0.14, 0.97) and with TPN (RR, 0.39; 95% CI, 0.26, 0.58). Furthermore, NG led to a significantly lower infected pancreatic necrosis rate when compared with TPN (RR, 0.31, 95% CI, 0.11, 0.9). The SUCRA showed that NG may be the optimal therapy (mean rank = 1.4; SUCRA = 86.8) for avoiding infected pancreatic necrosis, and NNS may be the least effective treatment, very likely resulting in infected pancreatic necrosis (mean rank = 3.5; SUCRA = 16.8) among patients with acute pancreatitis (Appendix D1-D4 in S1–File).

Bacteremia was reported in 7 RCTs.[6, 7, 10, 43, 45, 51, 52] The network meta-analysis showed no significant difference in bacteremia rates among the various therapies. However, the SUCRA showed that NNS may have the lowest probability of bacteremia (mean rank = 1.5; SUCRA = 82.2), whereas TPN may have the highest probability of leading to bacteremia (mean rank = 3.7; SUCRA = 9.8) among the 4 therapies (Appendix E1-E3 in S1 File).

Ten RCTs reported data on line infection.[6, 9–13, 40, 45, 51, 55] Because no RCT that compared NG with other therapies reported line infection, the consistency model was only conducted for comparisons among NNS, TPN, and NJ. The network meta-analysis showed that NJ had significantly lower line infection rates when compared with TPN (RR, 0.19; 95% CI, 0.07, 0.50). The SUCRA showed that NJ may have the lowest probability of leading to line infection (mean rank = 1.1; SUCRA = 94.3), and TPN may have the highest probability of leading to line infection (mean rank = 2.6; SUCRA = 20.9) among the 3 therapies (Appendix F1-F3 in S1 File).

Seven RCTs reported pneumonia.[9, 10, 12, 41, 43, 45, 52] The consistency model could only be conducted for comparisons among TPN, NJ, and NG, because no RCT that compared NNS with other therapies reported pneumonia. The network meta-analysis showed no significant difference in pneumonia rates among the 3 therapies. However, the SUCRA implied that NG may be the optimal therapy (mean rank = 1.1; SUCRA = 92.9) for avoiding pneumonia, and TPN may have the highest probability of leading to pneumonia (mean rank = 2.7; SUCRA = 15.8) among the 3 therapies (Appendix G1-G3 in S1 File).

Five RCTs reported data on urinary tract infections.[6, 9, 10, 12, 45] Because these 5 RCTs only compared NJ with TPN, no consistency model was required. We conducted a head-to-head meta-analysis for urinary tract infections, and results showed no significant difference in urinary tract infection rates between NJ and TPN (RR, 0.76; 95% CI, 0.27, 2.09) with very low heterogeneity (I-square = 0%) (Appendix H1 and H2 in S1 File).

Nine RCTs reported data on OTIs.[6–8, 13, 40, 43, 45, 52, 54] Because no RCT that compared NNS with other therapies reported OTIs, the consistency model was only conducted for comparisons among TPN, NJ, and NG. The results showed no significant difference in OTI rates among the 3 therapies. However, the SUCRA showed that NJ may have the lowest probability of leading to OTIs (mean rank = 1.7; SUCRA = 64.8), and TPN may have the highest probability of leading to OTIs (mean rank = 2.2; SUCRA = 37.8) among the 3 therapies (Appendix I1-I4 in S1 File).

## Discussion

This is the first network meta-analysis comparing infections among three nutritional support routes and NNS among acute pancreatitis. We summarized available data on overall infectious complications from 16 RCTs, and our analyses also detailed for six infectious events including infected pancreatic necrosis, bacteremia, line infection, pneumonia, urinary tract infections, and OTIs. Then, we found that NG may be the most preferred therapy and TPN may be the least preferred.

An important finding is about NG showing a favorable result in preventing infected pancreatic necrosis and pneumonia. To our knowledge, infected pancreatic necrosis was categorized as severe acute pancreatitis, and it may lead to morbidity and mortality.[57, 58] The superior ranking of enteral nutrition in our study may reflected that enteral feeding reduced bacterial translocation and further reducing colonization of the necrotic pancreatic tissue through keeping gut structure and function.[59] In contrast, NNS was the least effective therapy for preventing infected pancreatic necrosis because the hypercatabolic status and lack of nutritional support during long periods of illness lead to poor outcomes among pSAP patients.

Besides, we found another crucial piece of evidence for verifying the safety of using NG feeding in leading pneumonia. Aspiration is known to be an etiology of pneumonia, and in enteral nutrition, NG has long been believed to be more likely to cause aspiration than NJ.[60] However, our evidence did not support the assumption. The aforementioned evidence resonates with recent guidelines that support the efficacy and safety of NG and NJ in pSAP.[4, 15] The potential mechanism may be that pancreatic exocrine function was diminished significantly in patients with acute pancreatitis, and it has a negative correlation with the severity of acute pancreatitis.[61] As a result, NG is less likely to cause pancreatic stimulation and inflammation through an enzyme attack during a severe course. In the present study, although the result of NG being superior to NJ was nonsignificant, NG has numerous advantages. Specifically, it is much easier to insert, has a low dislodge rate, can achieve the same target caloric intake as NJ, and possesses the same risk of changing to TPN as NJ.[62]

Regarding the event of bacteremia, NNS is the most recommended therapy, NG is second, and TPN is also the least preferred. Two methodological problems may help to understand why NNS is the most recommended therapy. First, only one RCT was involved in the analysis.[51] Second, most participants in the RCT had mild acute pancreatitis. This result echoed current guidelines in terms of when treating patients with mild acute pancreatitis. NNS may be superior, because it may relieve patients’ symptoms rapidly.[63] However, the bacteremia rate in enteral nutrition is still lower than in TPN. Most studies that featured the event of bacteremia were on patients with pSAP,[6, 7, 10, 51, 52] and this implied the importance of enteral nutrition in such patients. This result supports the previous research indicating enteral nutrition prevents bacterial translocation.[64]

Our study further considered potential factors: severity and prophylactic antibiotics usage. Firstly, patients with pSAP are more likely to possess morbidities including pancreatic necrosis and infectious events.[58] We defined pSAP according to RCTs that mentioned the severity of acute pancreatitis,[6–13, 41–43, 50, 52–56] and NG seemed to be the preferred nutritional therapy among patients with pSAP. Secondly, physicians usually prescribed prophylactic antibiotics to patients with pSAP to prevent further infectious complications. In the further analysis of prophylactic antibiotics usage, the 9 included RCTs only recruited patients with pSAP. Most of them used imipenem and fluoroquinolone. Then, NG still seemed to be the most recommended treatment, and TPN was not recommended. We observed some interesting changes in effect size when comparing the results from 16 RCTs with the results from those providing all patients prophylactic antibiotics. The effect size of NG and TPN in the further analysis of prophylactic antibiotics (RR, 0.19) was lower than that in all RCT analyses (RR, 0.48). Moreover, the effect size of NG and NJ in the further analysis of prophylactic antibiotics (RR, 0.32) was also lower than that in all RCT analyses (RR, 0.82). These interesting findings implies that NG may be a superior choice, especially for those pSAP with prophylactic antibiotics.

### Limitations

This study has some limitations. Firstly, we could not control the etiology of acute pancreatitis. Etiology usually causes different infectious complication and require different treatments in acute pancreatitis. Regrettably, the RCTs included in this study did not differentiate etiologies. Thus, etiology might have influenced the results of this study. Secondly, the inclusion criteria about severity in the included RCTs used various scales, and the pSAP in this study was based the declaration in original study. We cannot exclude this potential bias from severity measurement. Thirdly, we did not consider the total caloric intake. Recent large trials,[65, 66] CALORIES and NUTRIREA-2, indicated that different caloric intakes may influence the outcome of infectious complications. The 22 included RCTs set different caloric goals and caloric achievements. Caloric intake was another difficult-to-control confounding factor. Fourthly, we did not get any response from authors for missing data. That is to say, our results still cannot completely reflect all evidence because of those missing data though our evidence is the first network meta-analysis of the effects nutritional routes on acute pancreatitis. Lastly, SUCRA was regarded to have a substantial degree of imprecision in ranking.[67] Nevertheless, in our study, the effect size and probability ranking showed obvious trends in the outcomes, except for in OTIs. These clear ranks provide clear and practical information. Notably, these limitations were not well-controlled in previous systematic reviews either.

## Conclusions

In conclusion, acute pancreatitis is an inflammatory disease with unregulated activation of trypsin in pancreatic acinar cells. Our evidence echoed that the era of “gut rousing” replaced “pancreatic rest”.[68] To prevent further infectious complications, selecting an adequate nutritional support is crucial. Overall, NG was shown to be the most preferred therapy for acute pancreatitis and TPN was shown to be the least preferred. Moreover, NNS must be avoided in treating patients with severe acute pancreatitis. More evidence is required to further analyze the etiology, feeding time, and caloric intake in acute pancreatitis.

## Supporting information

S1 File Further analysis. (PDF)

S2 File PRISMA checklist. (PDF)

S1 Table Database and search strategy. (PDF)

S2 Table Risk of bias. (PDF)

S3 Table Further information of the included randomized clinical trials. (PDF)

S4 Table Regression by inclusion year and total parenteral nutrition timing. (PDF)

S5 Table Summary of surface under the cumulative ranking curve analysis. (PDF)

## Abbreviations

AP: acute pancreatitis
CI: confidence interval
NG: nasogastric
NJ: nasojejunal
NNS: no nutritional support
OTI: other type of infection
pSAP: predicted severe acute pancreatitis
RCT: randomized control trial
RR: risk ratio
SUCRA: surface under the cumulative ranking curve
TPN: total parenteral nutrition
