# Supplementary material for: Infection rate among nutritional therapies for acute pancreatitis: A systematic review with network meta-analysis of randomized controlled trials
Source: PLoS One. 2019 Jul 10;14(7):e0219151. doi: 10.1371/journal.pone.0219151 (PMC6620007; doi:10.1371/journal.pone.0219151)
Supplement: S1 File — (PDF) [file pone.0219151.s001.pdf]

Infection Rate Among Nutritional Therapies for Acute Pancreatitis:

A Systematic Review with Network Meta-Analysis of Randomized Controlled Trials

S1 File. Further analysis

Index of Appendix

Appendix A1. Pairwise comparison and SUCRA of total infection rates

Appendix A2. Inconsistency test for network meta-analysis of total infection rates

Appendix A3. Small study bias in network meta-analysis of total infection rates

Appendix B1. Network geometry of total infection rates (pSAP)

Appendix B2. Ranking probability and SUCRA of total infection rates (pSAP)

Appendix B3. Inconsistency test for network meta-analysis of total infection rates (pSAP)

Appendix B4. Small study bias in network meta-analysis of total infection rates (pSAP)

Appendix C1. Network geometry of total infection rates (PAB)

Appendix C2. Ranking probability and SUCRA of total infection rates (PAB)

Appendix C3. Inconsistency test for network meta-analysis of total infection rates (PAB)

Appendix C4. Small study bias in network meta-analysis of total infection rates (PAB)

Appendix D1. Network geometry of infected pancreatic necrosis

Appendix D2. Ranking probability and SUCRA of infected pancreatic necrosis

Appendix D3. Inconsistency test for network meta-analysis of infected pancreatic necrosis

Appendix D4. Small study bias in network meta-analysis of infected pancreatic necrosis

Appendix E1. Network geometry of bacteremia

Appendix E2. Ranking probability and SUCRA of bacteremia

Appendix E3. Small study bias in network meta-analysis of bacteremia

Appendix F1. Network geometry of line infection

Appendix F2. Ranking probability and SUCRA of line infection

Appendix F3. Small study bias in network meta-analysis of line infection

Appendix G1. Network geometry of pneumonia

Appendix G2. Ranking probability and SUCRA of pneumonia

Appendix G3. Small study bias in network meta-analysis of pneumonia

Appendix H1. Pairwise meta-analysis of urinary tract infection

Appendix H2. Small study bias in pairwise meta-analysis of urinary tract infection

Appendix I1. Network geometry of other type infection

Appendix I2. Ranking probability and SUCRA of other type infection

Appendix I3. Inconsistency test for network meta-analysis of other type infection

Appendix I4. Small study bias in network meta-analysis of other type infection

Appendix A1 to C4
Outcomes of total infection rates

Appendix A1

Pairwise comparison and SUCRA of total infection rates

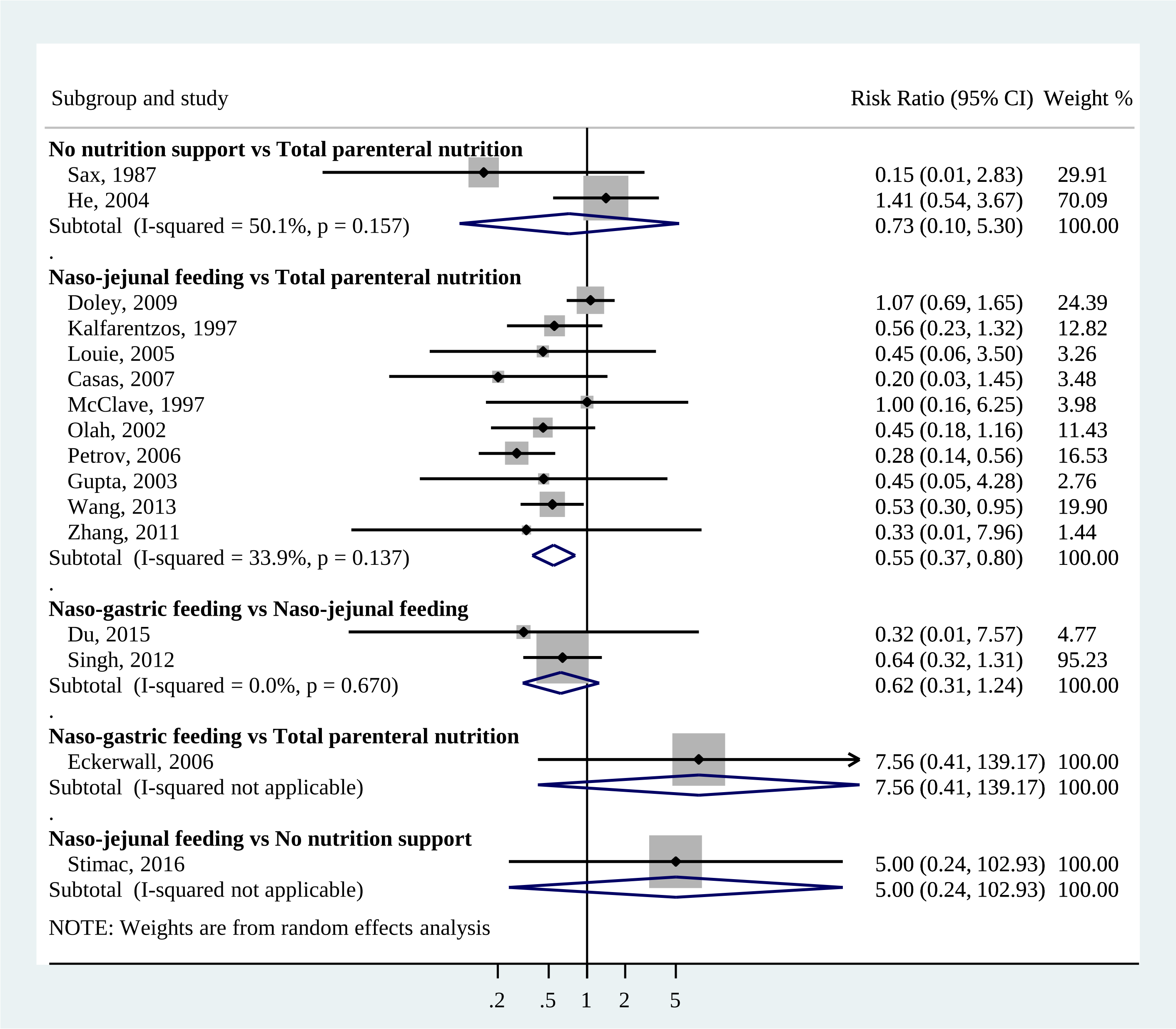

. sucra prob*, labels (TPN NNS NJ NG)

Treatm~t	SUCRA	PrBest	MeanRank
TPN	14.5	0.1	3.6
NNS	42.2	20.7	2.7
NJ	67.5	23.3	2.0
NG	75.8	55.8	1.7

NG, naso-gastric; NJ, naso-jejeunal; NNS, no nutrition support; TPN, total parenteral nutrition.

Appendix A2

Inconsistency test for network meta-analysis of total infection rates

```
. network meta i, luades

initial:       log likelihood = -33.925748
rescale:       log likelihood = -32.902751
rescale eq:    log likelihood = -31.792065
Iteration 0:   log likelihood = -31.792065
Iteration 1:   log likelihood =  -31.63377
Iteration 2:   log likelihood = -31.596862
Iteration 3:   log likelihood = -31.596587
Iteration 4:   log likelihood = -31.596587

Multivariate meta-analysis
Method = reml                          Number of dimensions    =     3
Restricted log likelihood = -31.596587       Number of observations  =    16
------------------------------------------------------------------------------
             |      Coef.   Std. Err.      z    P>|z|     [95% Conf. Interval]
-------------+----------------------------------------------------------------
_y_B         |
       _cons |   .0279795   .5848322     0.05   0.962    -1.118271     1.17423
-------------+----------------------------------------------------------------
_y_C         |
      groupB |   2.258552   1.707176     1.32   0.186    -1.087451    5.604556
       _cons |  -.6211678   .2180676    -2.85   0.004    -1.048572   -.1937631
-------------+----------------------------------------------------------------
_y_D         |
      groupC |  -3.151118   1.633009    -1.93   0.054    -6.351756    .0495203
       _cons |   2.022871   1.536149     1.32   0.188    -.9879256    5.033668
------------------------------------------------------------------------------

Estimated between-studies SDs and correlation matrix:
             SD        _y_B        _y_C        _y_D
_y_B  .38868595           1           .           .
_y_C  .38868595          .5           1           .
_y_D  .38868595          .5          .5           1

Testing for inconsistency:
 ( 1)  [_y_C]groupB = 0
 ( 2)  [_y_D]groupC = 0

           chi2(  2) =    5.40
         Prob > chi2 =    0.0673
```

Appendix A3

Small study bias in network meta-analysis of total infection rates

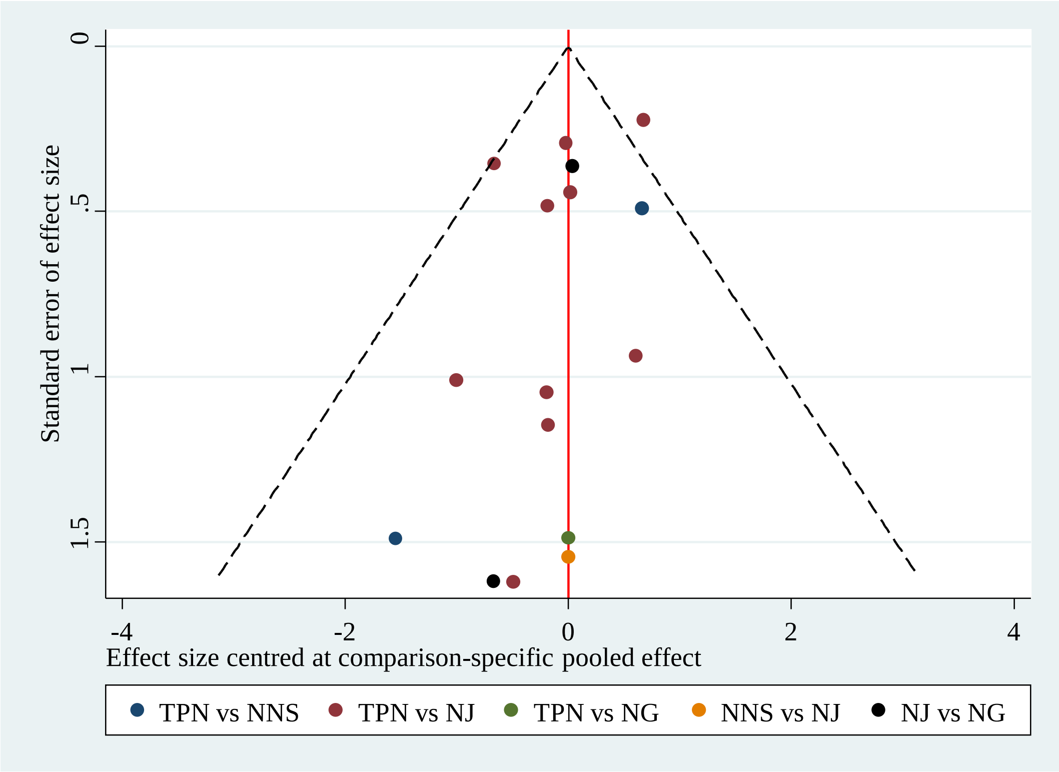

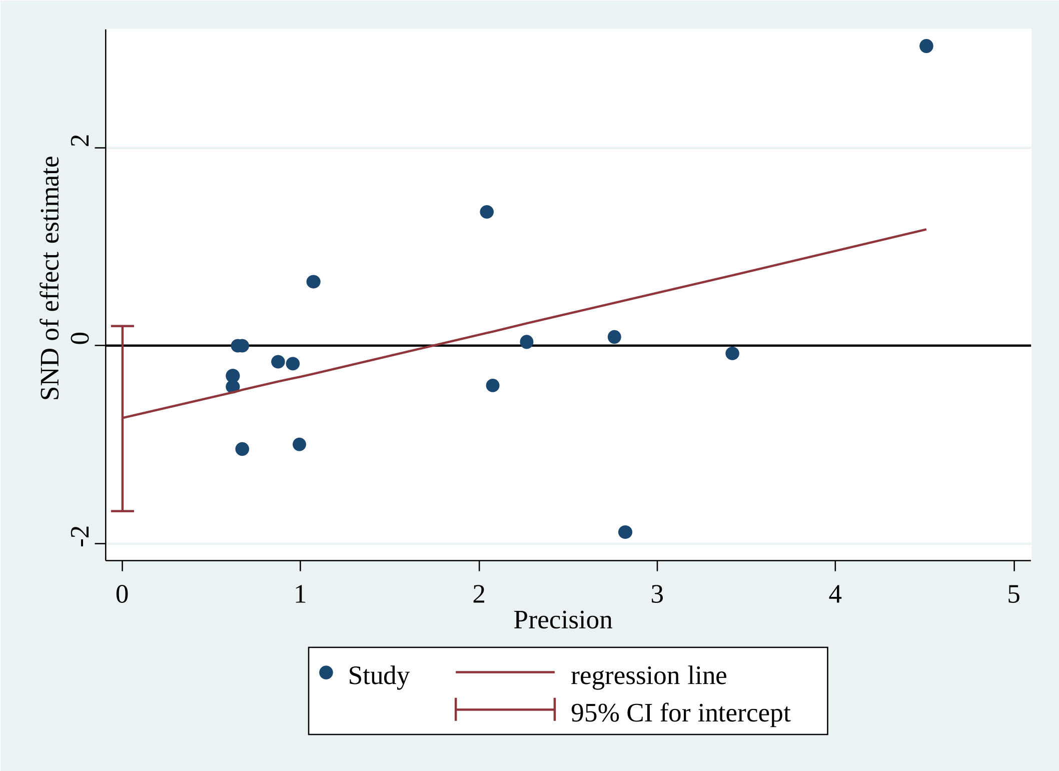

NG, naso-gastric; NJ, naso-jejeunal; NNS, no nutrition support; TPN, total parenteral nutrition.

Appendix B1

Network geometry of total infection rates (pSAP)

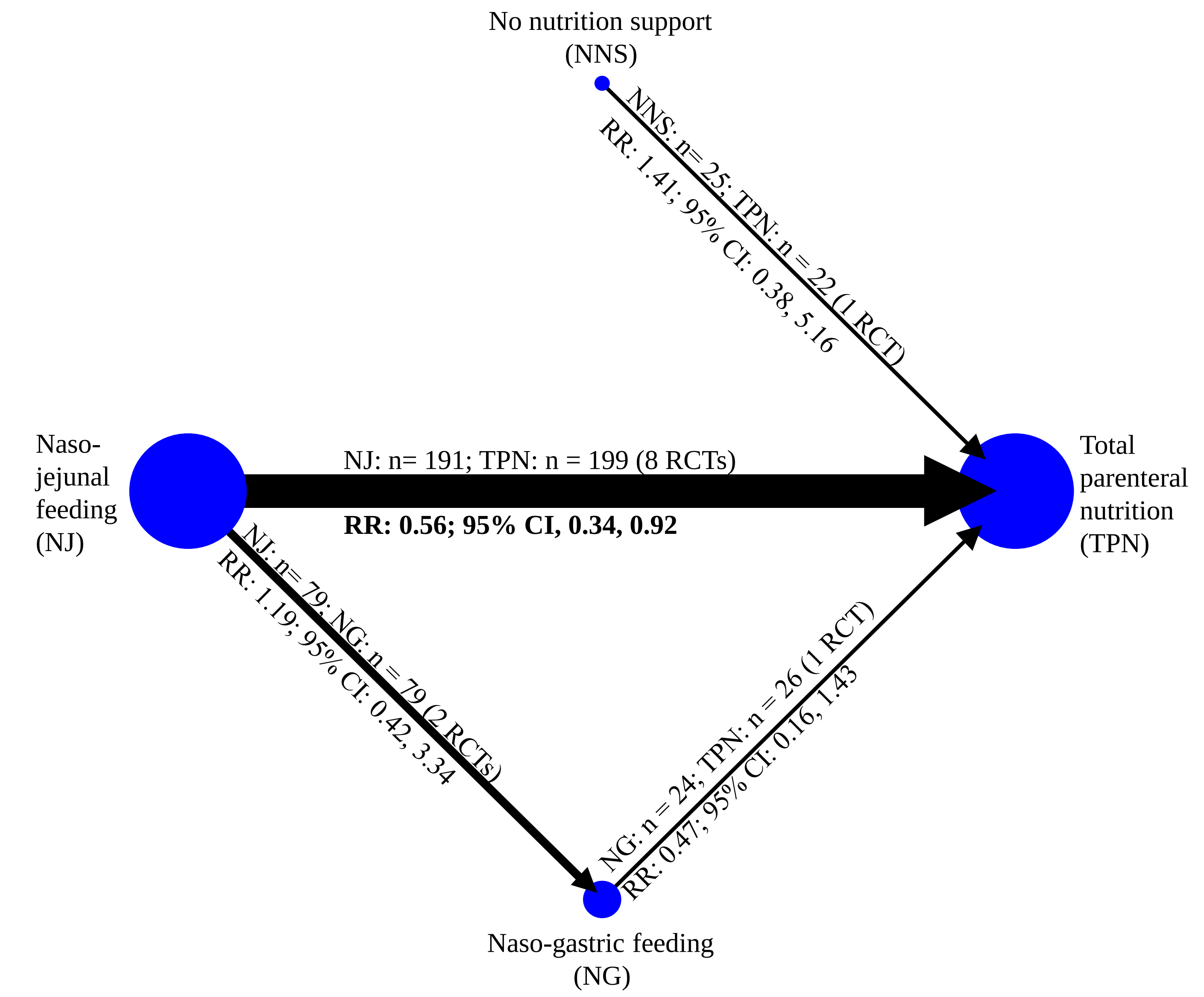

NG, naso-gastric; NJ, naso-jejeunal; NNS, no nutrition support; TPN, total parenteral nutrition.

Appendix B2

Ranking probability and SUCRA of total infection rates (pSAP)

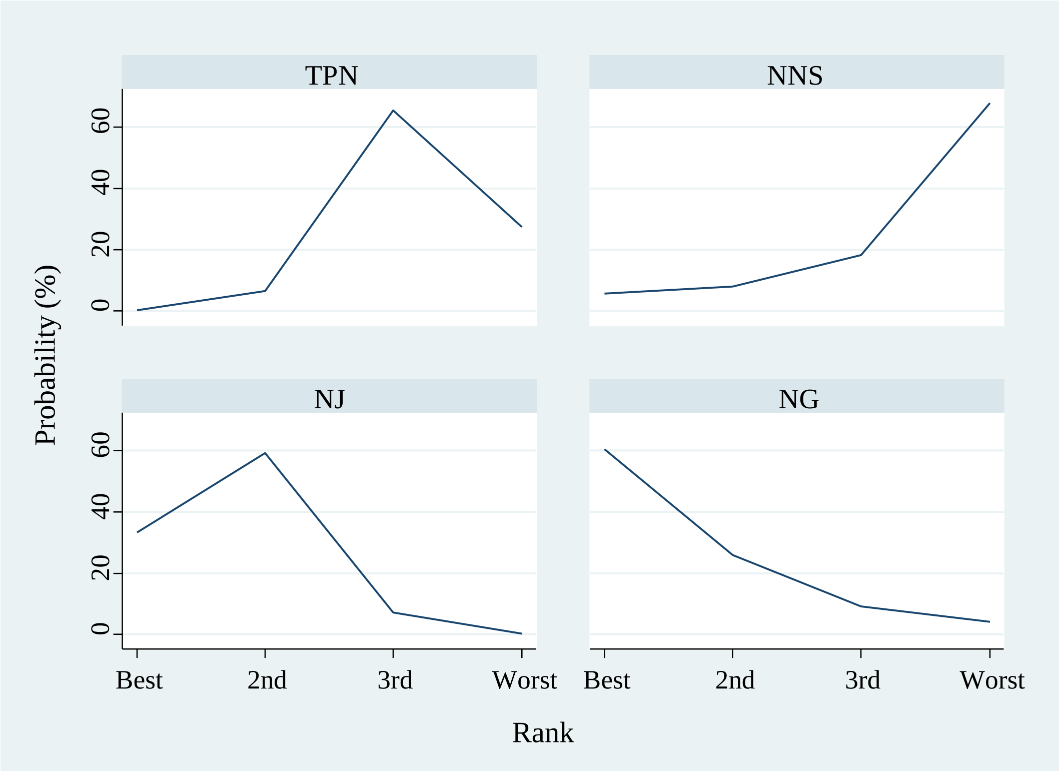

. sucra prob*, labels (TPN NNS NJ NG)

Treatm~t	SUCRA	PrBest	MeanRank
TPN	26.6	0.3	3.2
NNS	17.3	5.8	3.5
NJ	75.2	33.4	1.7
NG	80.9	60.5	1.6

NG, naso-gastric; NJ, naso-jejeunal; NNS, no nutrition support; TPN, total parenteral nutrition.

Appendix B3

Inconsistency test for network meta-analysis of total infection rates (pSAP)

```
. network meta i, luades

initial:       log likelihood = -22.693079
rescale:       log likelihood = -21.991527
rescale eq:    log likelihood = -21.175275
Iteration 0:   log likelihood = -21.175275
Iteration 1:   log likelihood = -21.072764
Iteration 2:   log likelihood = -21.063997
Iteration 3:   log likelihood = -21.063974
Iteration 4:   log likelihood = -21.063974

Multivariate meta-analysis
Method = reml                                   Number of dimensions    =     3
Restricted log likelihood = -21.063974          Number of observations  =    12
------------------------------------------------------------------------------
             |      Coef.   Std. Err.      z    P>|z|     [95% Conf. Interval]
-------------+----------------------------------------------------------------
_y_B         |
       _cons |   .3421703   .6523271     0.52   0.600    -.9363674    1.620708
-------------+----------------------------------------------------------------
_y_C         |
       _cons |  -.6430696   .2555097    -2.52   0.012    -1.143859   -.1422798
-------------+----------------------------------------------------------------
_y_D         |
      groupC |  -3.179444   1.658393    -1.92   0.055    -6.429834    .0709461
       _cons |   2.022871    1.54747     1.31   0.191    -1.010114    5.055856
------------------------------------------------------------------------------

Estimated between-studies SDs and correlation matrix:
             SD        _y_B        _y_C        _y_D
_y_B  .43126002           1           .           .
_y_C  .43126002          .5           1           .
_y_D  .43126002          .5          .5           1

Testing for inconsistency:
 ( 1)  [_y_D]groupC = 0

           chi2(  1) =    3.68
         Prob > chi2 =    0.0552
```

Appendix B4

Small study bias in network meta-analysis of total infection rates (pSAP)

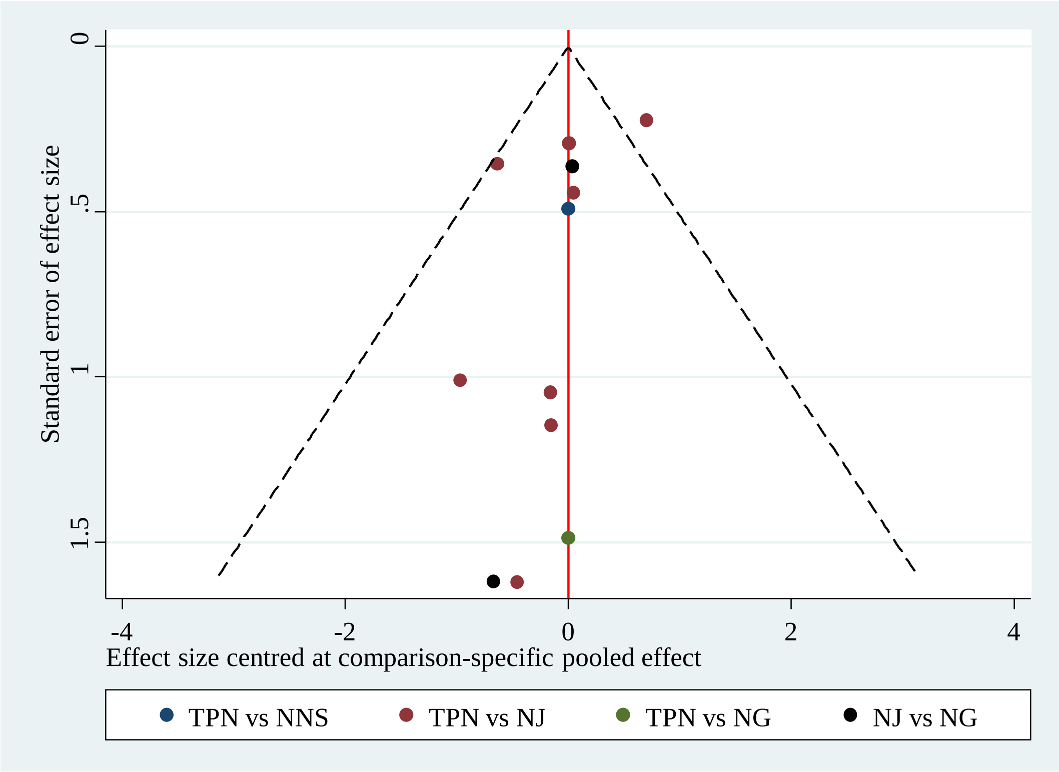

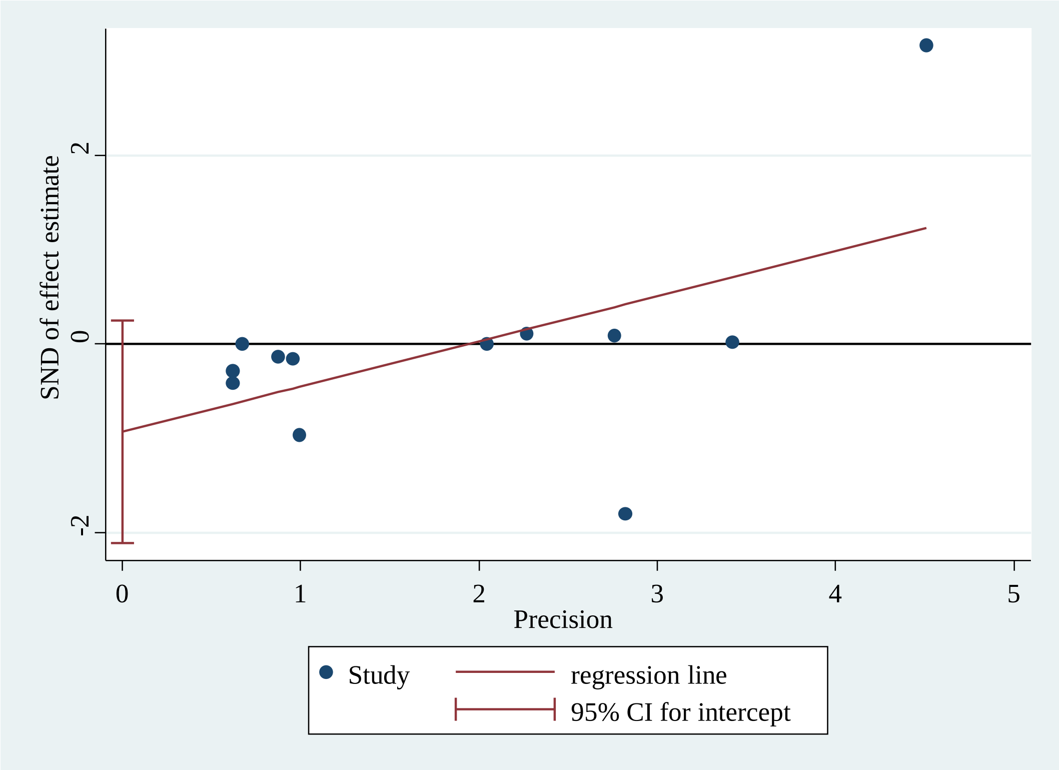

NG, naso-gastric; NJ, naso-jejeunal; NNS, no nutrition support; TPN, total parenteral nutrition.

Appendix C1

Network geometry of total infection rates (PAB)

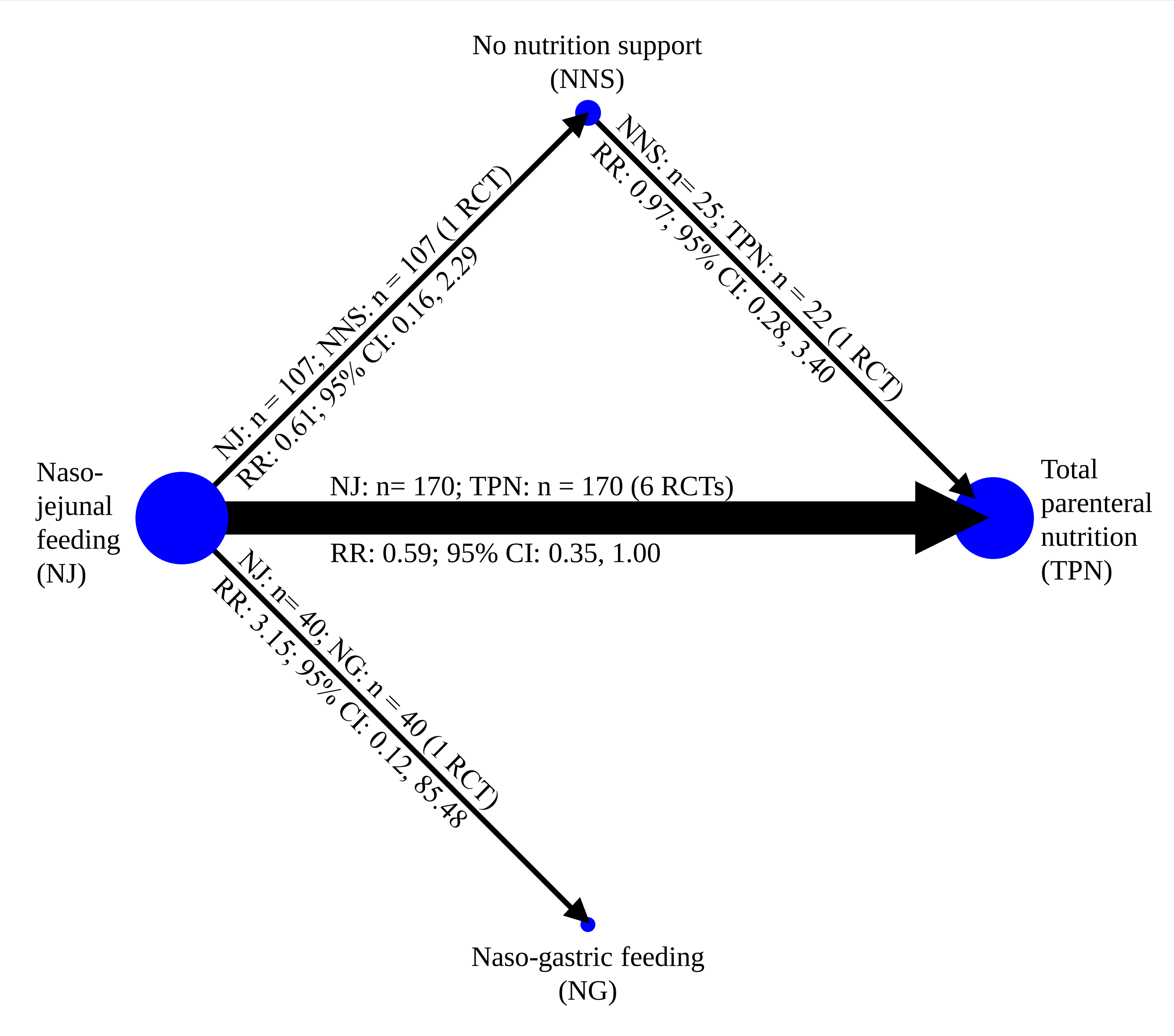

Appendix C2

Ranking probability and SUCRA of total infection rates (PAB)

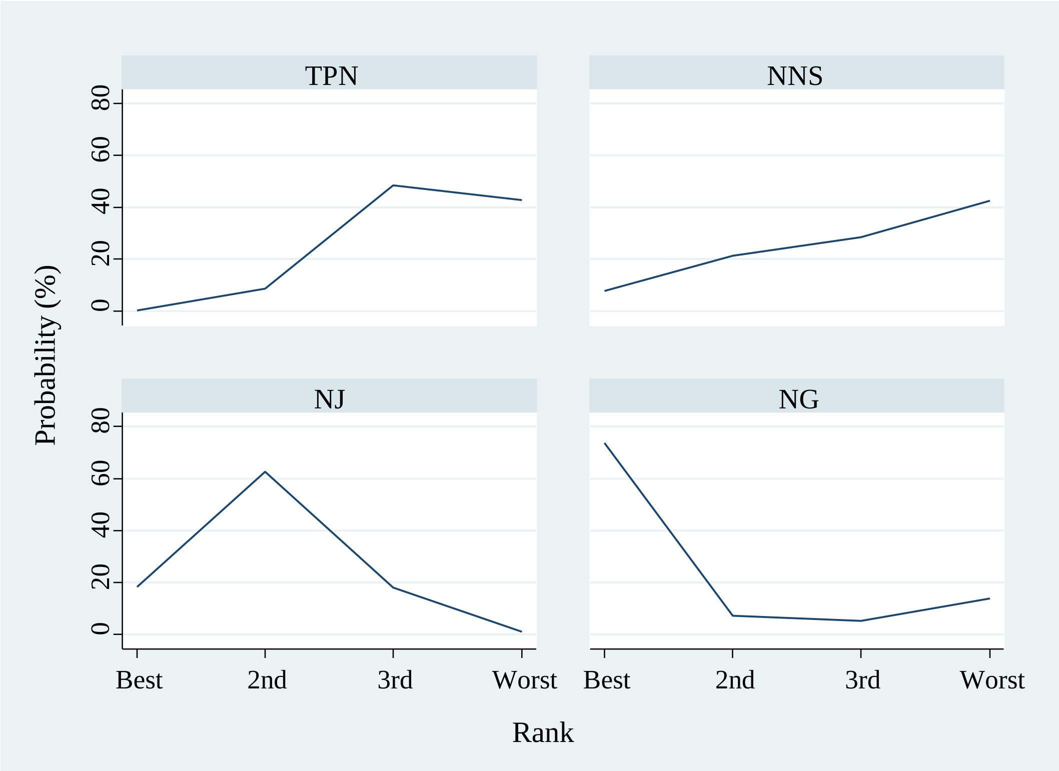

. sucra prob*, labels(TPN NNS NJ NG)

Treatm~t	SUCRA	PrBest	MeanRank
TPN	22.3	0.3	3.3
NNS	31.4	7.8	3.1
NJ	66.1	18.3	2.0
NG	80.2	73.6	1.6

NG, naso-gastric; NJ, naso-jejeunal; NNS, no nutrition support; TPN, total parenteral nutrition.

Appendix C3

Inconsistency test for network meta-analysis of total infection rates (PAB)

```
. network meta i, luades

initial:       log likelihood = -19.770755
rescale:       log likelihood = -19.327343
rescale eq:    log likelihood = -18.686484
Iteration 0:   log likelihood = -18.686484
Iteration 1:   log likelihood = -18.654607
Iteration 2:   log likelihood = -18.653687
Iteration 3:   log likelihood = -18.653687

Multivariate meta-analysis
Method = reml                              Number of dimensions    =     3
Restricted log likelihood = -18.653687        Number of observations  =     9
------------------------------------------------------------------------------
             |      Coef.   Std. Err.      z    P>|z|     [95% Conf. Interval]
-------------+----------------------------------------------------------------
_y_B         |
       _cons |   .3421612   .6689198     0.51   0.609    -.9688975     1.65322
-------------+----------------------------------------------------------------
_y_C         |
      groupB |   2.537296   1.763934     1.44   0.150    -.9199503    5.994543
       _cons |  -.5857398   .2733219    -2.14   0.032    -1.121441   -.0500387
-------------+----------------------------------------------------------------
_y_D         |
       _cons |  -1.733123   1.703073    -1.02   0.309    -5.071084    1.604838
------------------------------------------------------------------------------

Estimated between-studies SDs and correlation matrix:
             SD        _y_B        _y_C        _y_D
_y_B  .45597266           1           .           .
_y_C  .45597266          .5           1           .
_y_D  .45597266          .5          .5           1

Testing for inconsistency:
 ( 1)  [_y_C]groupB = 0

           chi2(  1) =    2.07
         Prob > chi2 =    0.1503
```

Appendix C4

Small study bias in network meta-analysis of total infection rates (PAB)

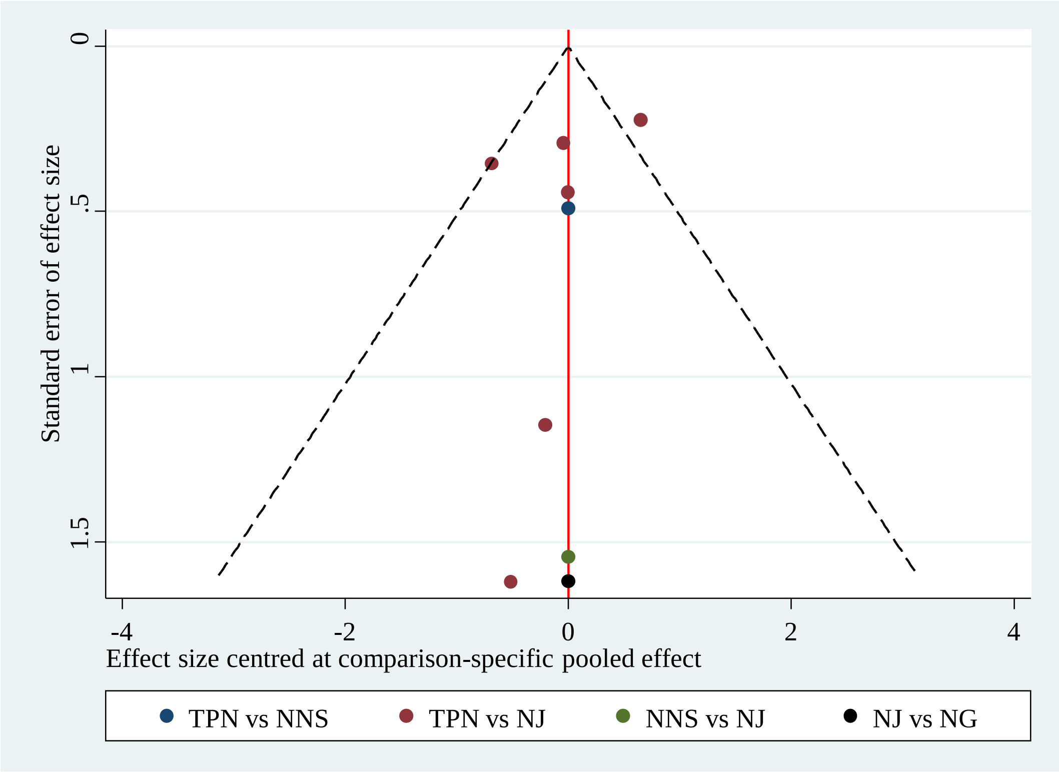

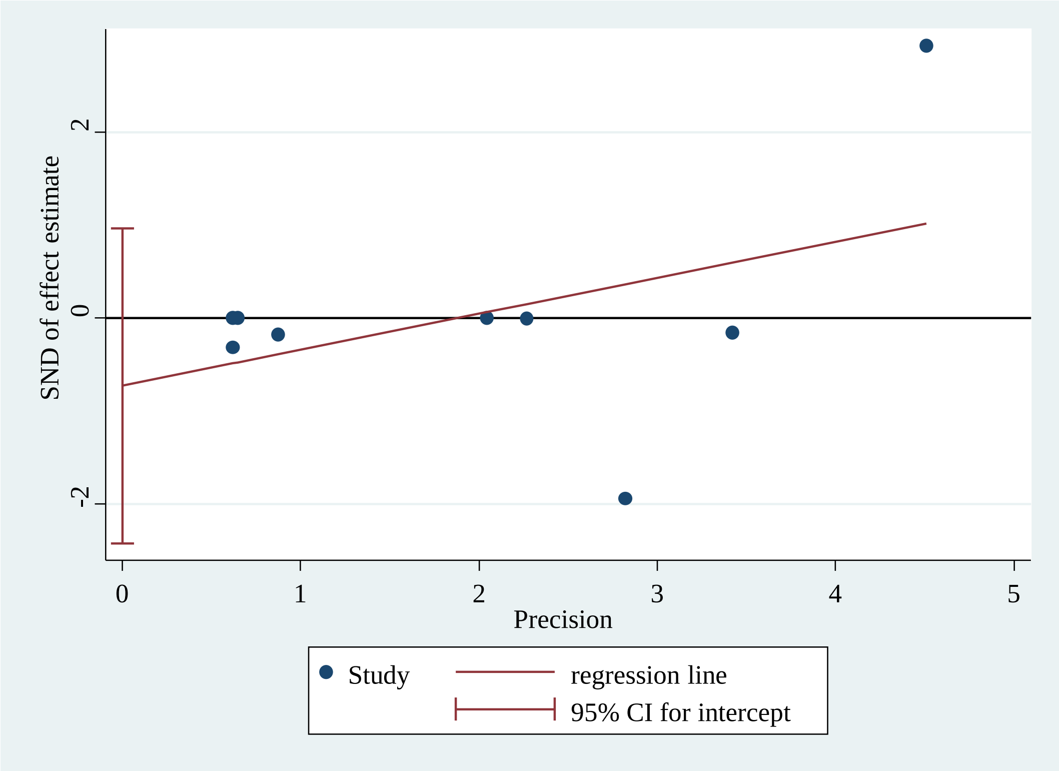

NG, naso-gastric; NJ, naso-jejeunal; NNS, no nutrition support; TPN, total parenteral nutrition.

Appendix D1 to D4
Outcomes of infected pancreatic necrosis

Appendix D1

Network geometry of infected pancreatic necrosis

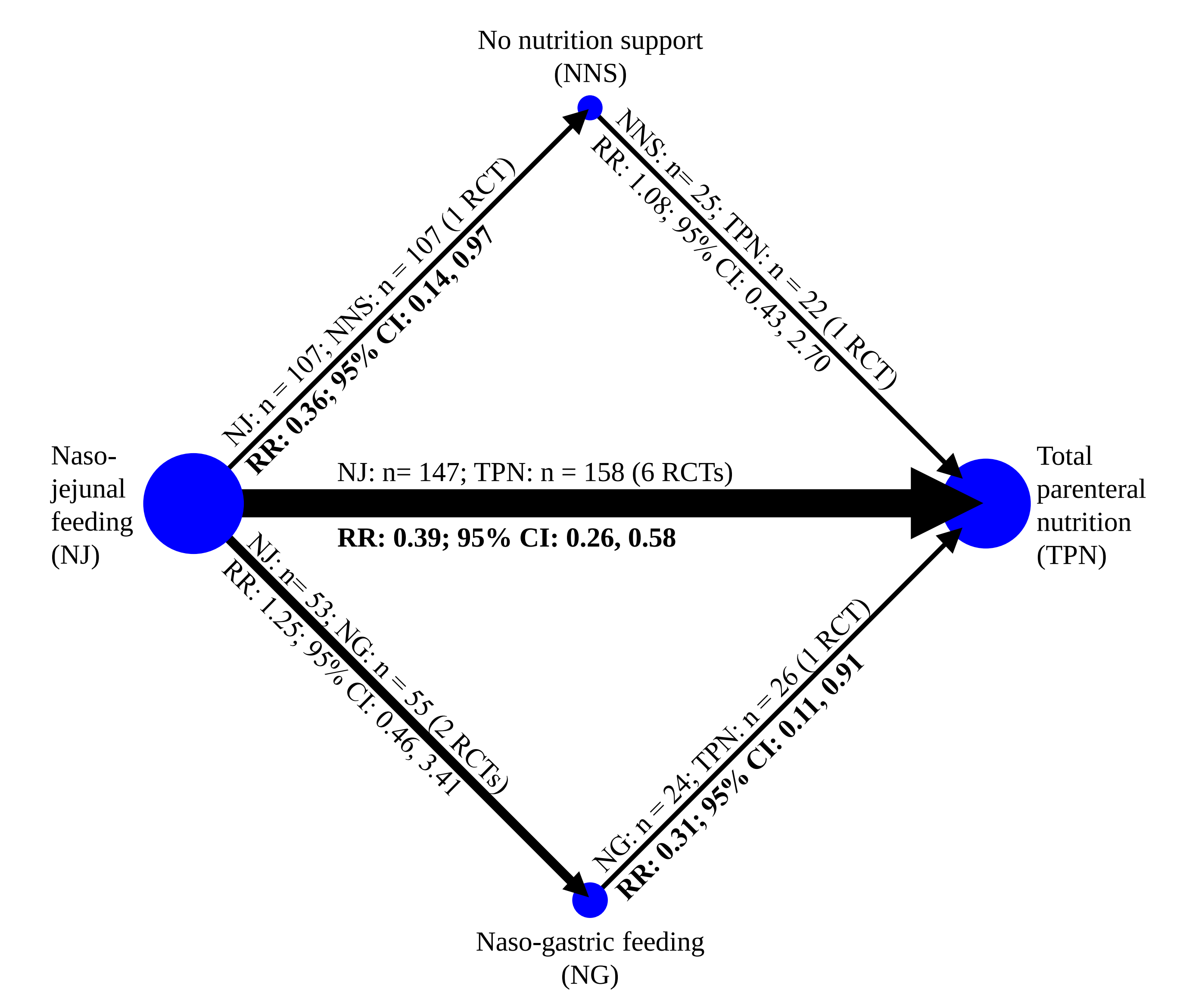

NG, naso-gastric; NJ, naso-jejeunal; NNS, no nutrition support; TPN, total parenteral nutrition.

Appendix D2

Ranking probability and SUCRA of infected pancreatic necrosis

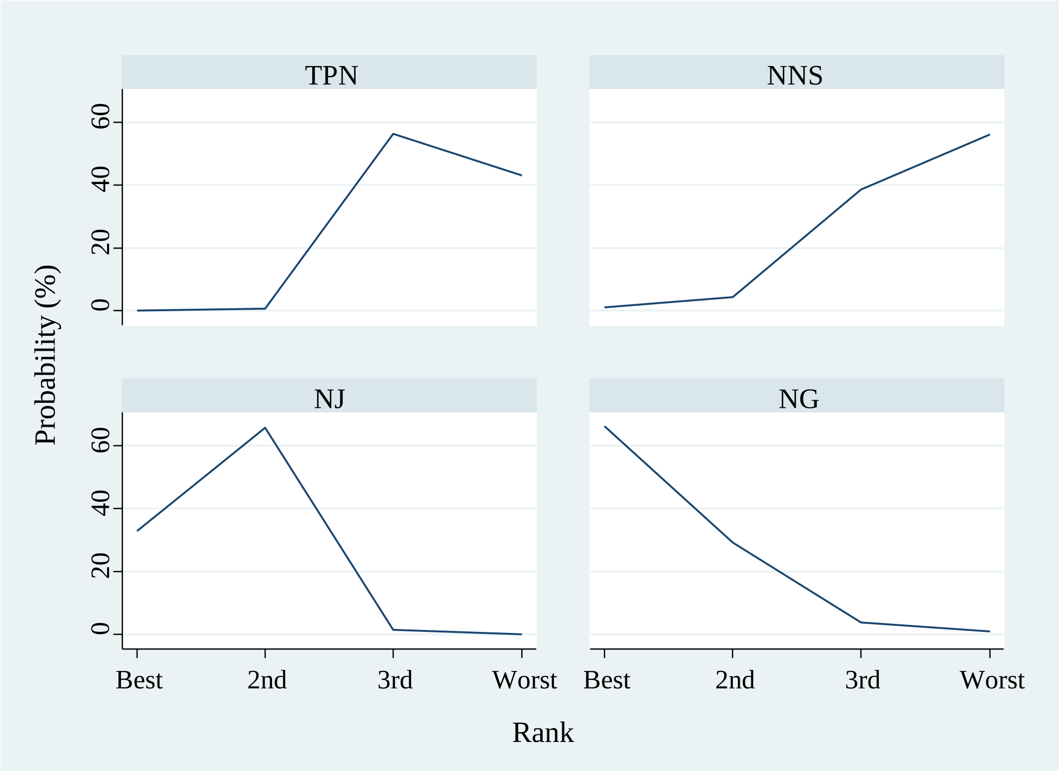


```
. sucra prob*, labels(TPN NNS NJ NG)

  +-------------------------------------+
  | Treatm~t | SUCRA | PrBest | MeanRank |
  |----------+-------+--------+----------|
  |      TPN |  19.3 |    0.0 |      3.4 |
  |      NNS |  16.8 |    1.0 |      3.5 |
  |       NJ |  77.1 |   32.8 |      1.7 |
  |       NG |  86.8 |   66.2 |      1.4 |
  +-------------------------------------+
```

NG, naso-gastric; NJ, naso-jejeunal; NNS, no nutrition support; TPN, total parenteral nutrition.

Appendix D3

Inconsistency test for network meta-analysis of infected pancreatic necrosis

```
. network meta i, luades

initial:       log likelihood = -26.007924
rescale:       log likelihood = -25.676174
rescale eq:    log likelihood = -23.538287
Iteration 0:   log likelihood = -23.538287
Iteration 1:   log likelihood = -23.492178
Iteration 2:   log likelihood = -23.492177

Multivariate meta-analysis
Method = reml                                   Number of dimensions     =      3
Restricted log likelihood = -23.492177          Number of observations   =     11
------------------------------------------------------------------------------
             |      Coef.   Std. Err.      z    P>|z|     [95% Conf. Interval]
-------------+----------------------------------------------------------------
_y_B         |
       _cons |   .3421654    .489433     0.70   0.484    -.6171056    1.301436
-------------+----------------------------------------------------------------
_y_C         |
      groupB |   2.974529   1.631749     1.82   0.068      -.22364    6.172698
       _cons |  -1.022966   .2040773    -5.01   0.000     -1.42295    -.622982
-------------+----------------------------------------------------------------
_y_D         |
      groupC |  -2.684975   1.709908    -1.57   0.116    -6.036333    .6663836
       _cons |   1.175573   1.609233     0.73   0.465    -1.978465    4.329612
------------------------------------------------------------------------------

Estimated between-studies SDs and correlation matrix:
             SD        _y_B        _y_C        _y_D
_y_B  1.855e-09           1           .           .
_y_C  1.855e-09          .5           1           .
_y_D  1.855e-09          .5          .5           1

Testing for inconsistency:
 ( 1)  [_y_C]groupB = 0
 ( 2)  [_y_D]groupC = 0

           chi2(  2) =    5.70
         Prob > chi2 =    0.0577
```

Appendix D4

Small study bias in network meta-analysis of infected pancreatic necrosis

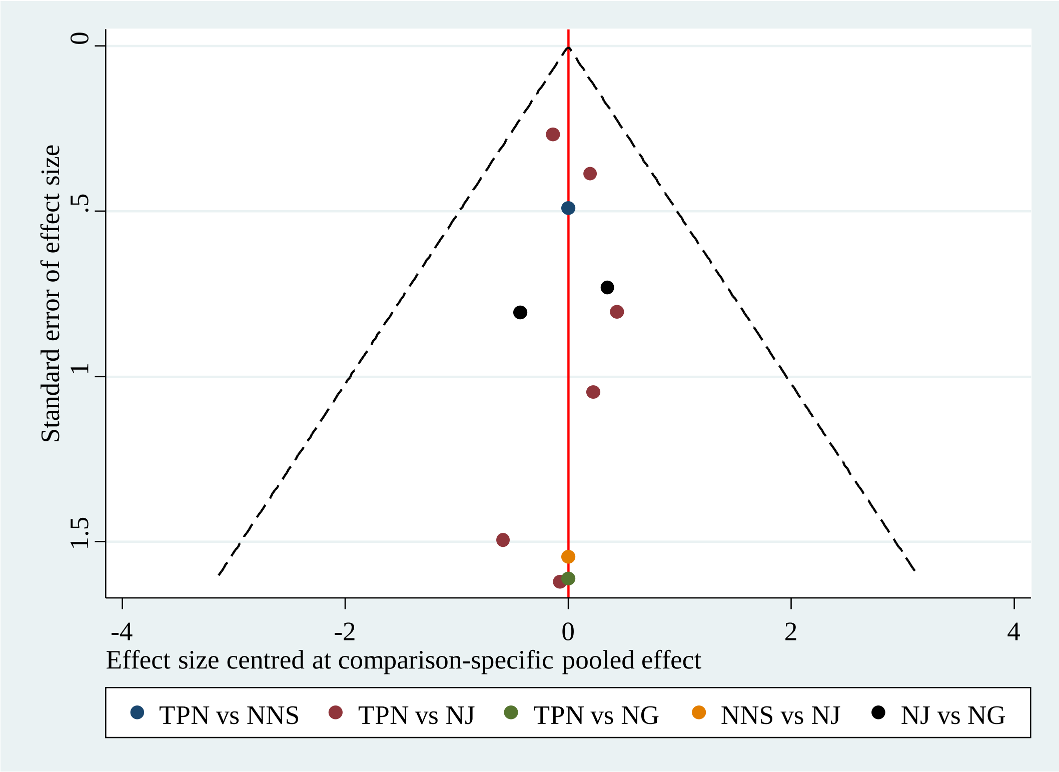

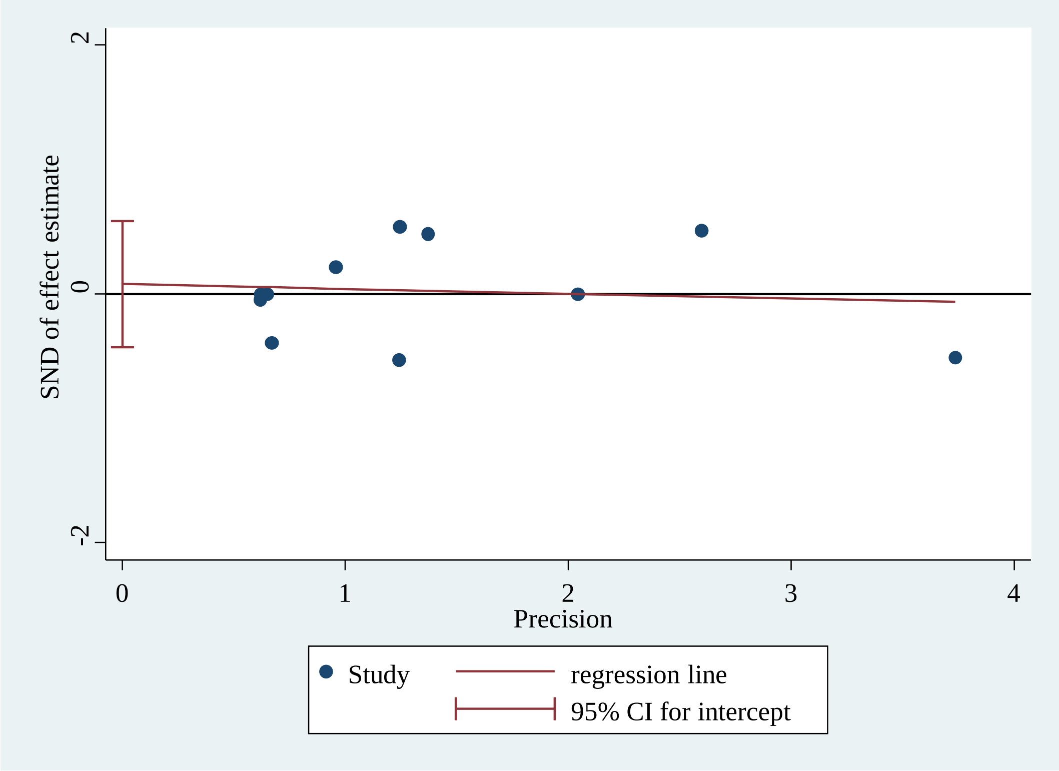

NG, naso-gastric; NJ, naso-jejeunal; NNS, no nutrition support; TPN, total parenteral nutrition.

Appendix E1 to E3
Outcomes of bacteremia

Appendix E1

Network geometry of bacteremia

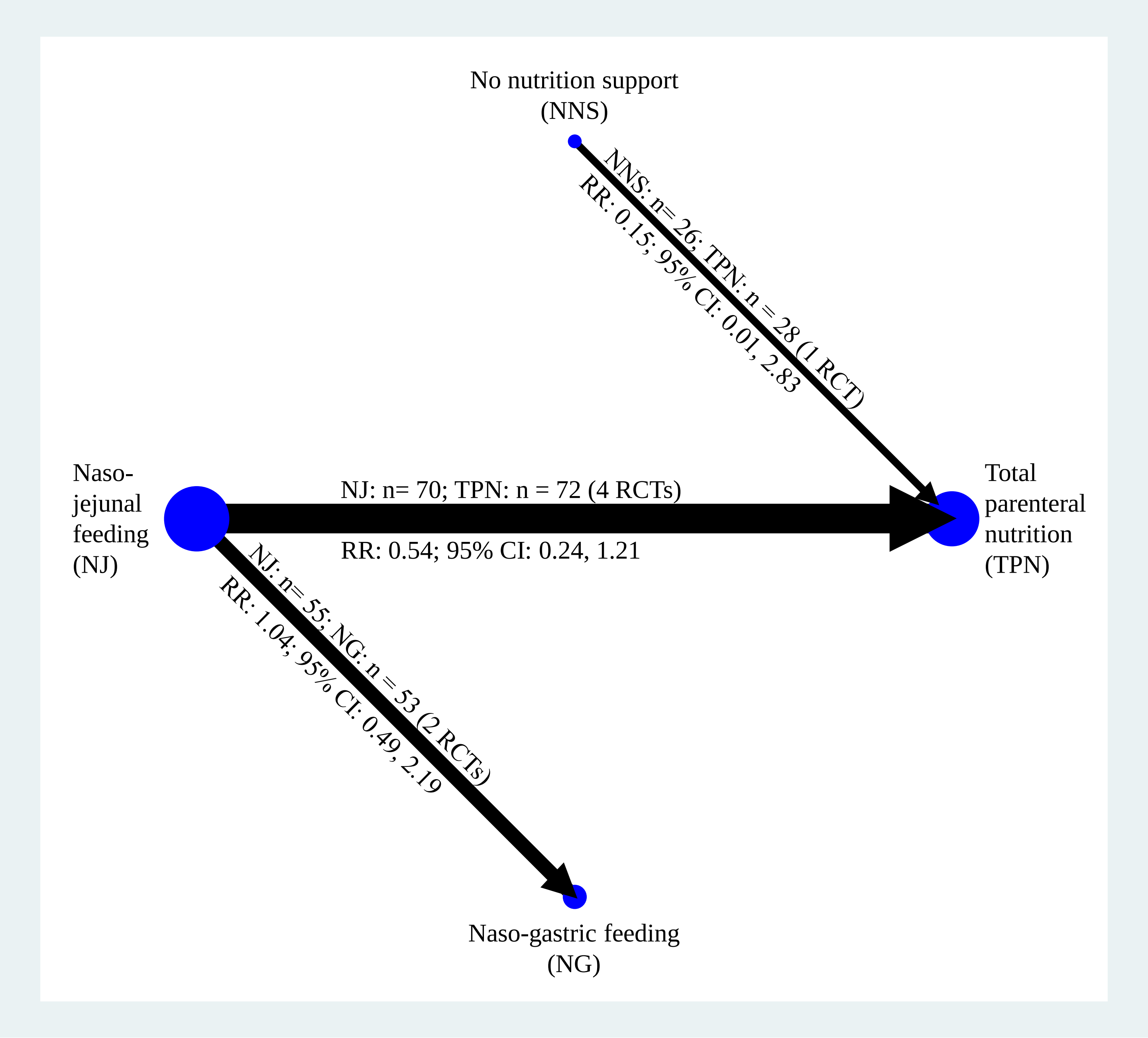

NG, naso-gastric; NJ, naso-jejeunal; NNS, no nutrition support; TPN, total parenteral nutrition.

Appendix E2
Ranking probability and SUCRA of bacteremia

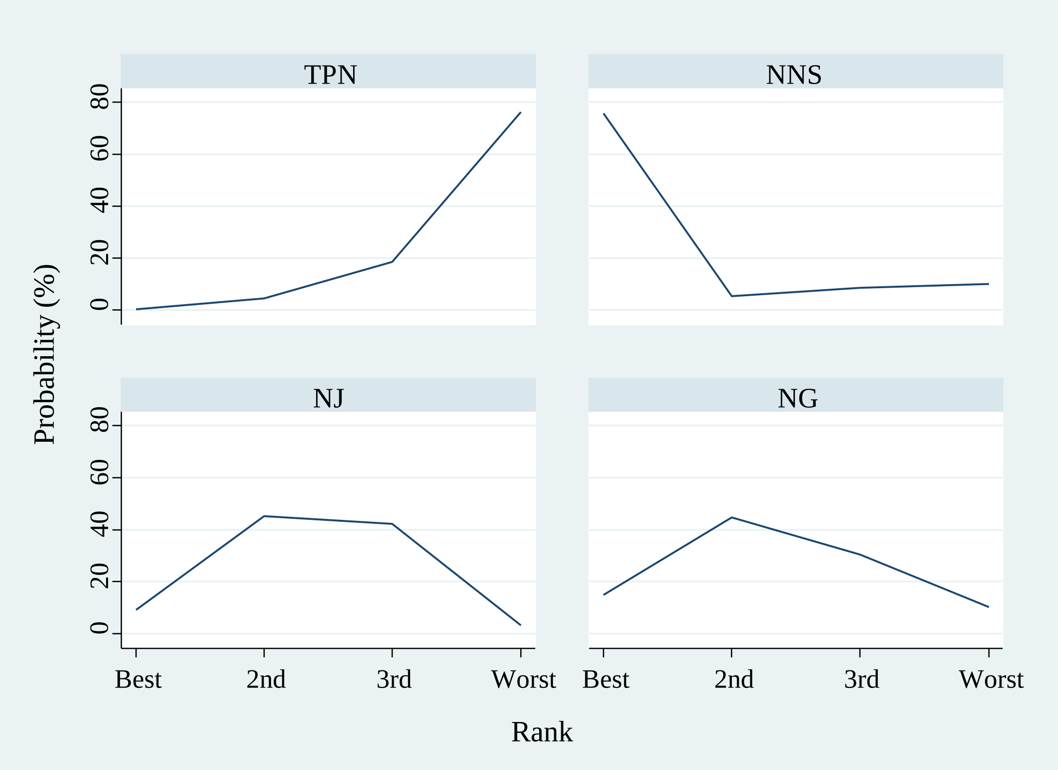

. sucra prob*, labels(TPN NNS NJ NG)

Treatm~t	SUCRA	PrBest	MeanRank
TPN	9.8	0.4	3.7
NNS	82.2	75.6	1.5
NJ	53.4	9.1	2.4
NG	54.7	14.8	2.4

NG, naso-gastric; NJ, naso-jejeunal; NNS, no nutrition support; TPN, total parenteral nutrition.

Appendix E3
Small study bias in network meta-analysis of bacteremia

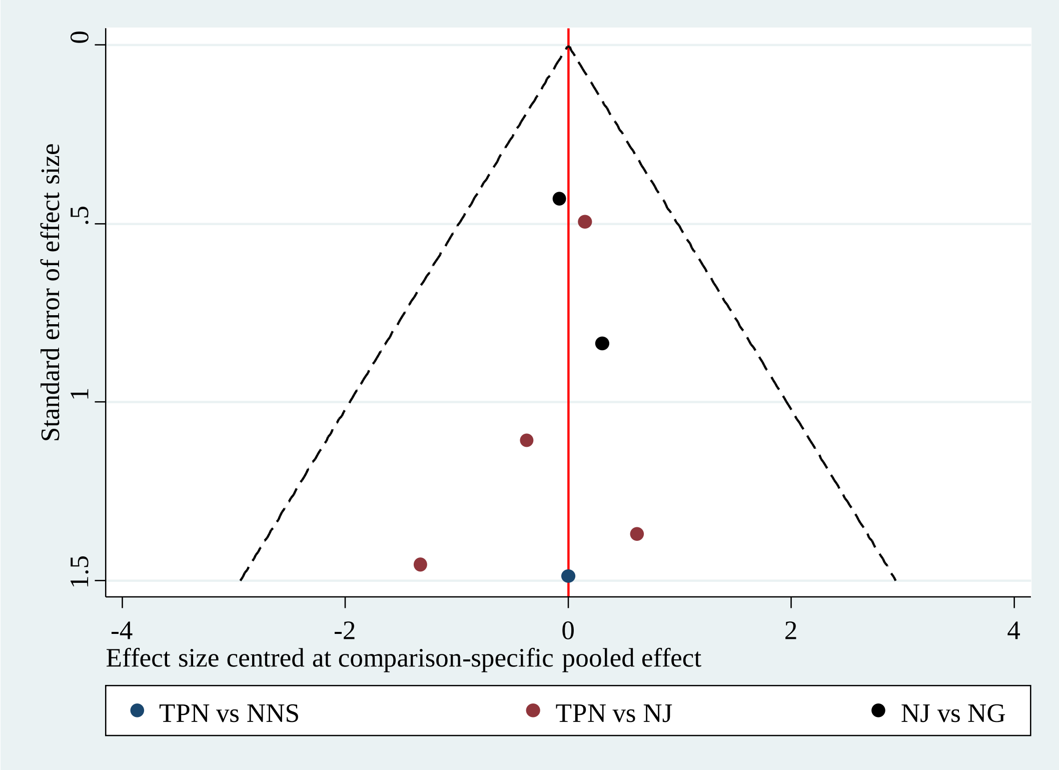

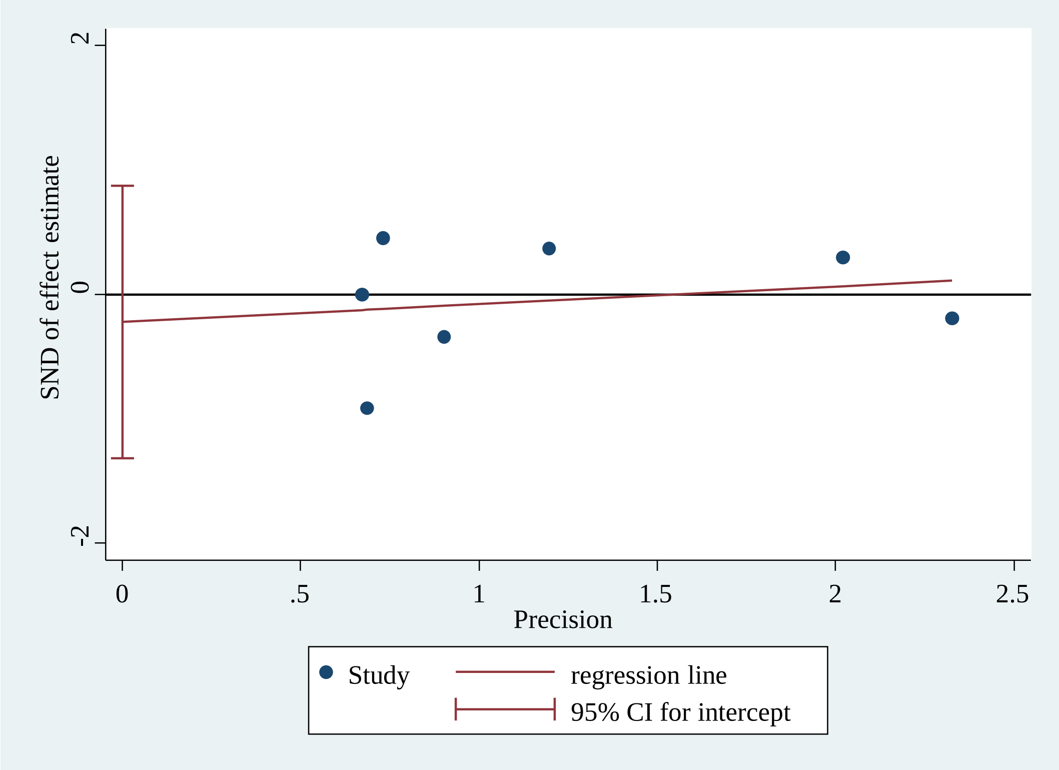

NG, naso-gastric; NJ, naso-jejeunal; NNS, no nutrition support; TPN, total parenteral nutrition.

Appendix F1 to F3
Outcomes of line infection

Appendix F1

Network geometry of line infection

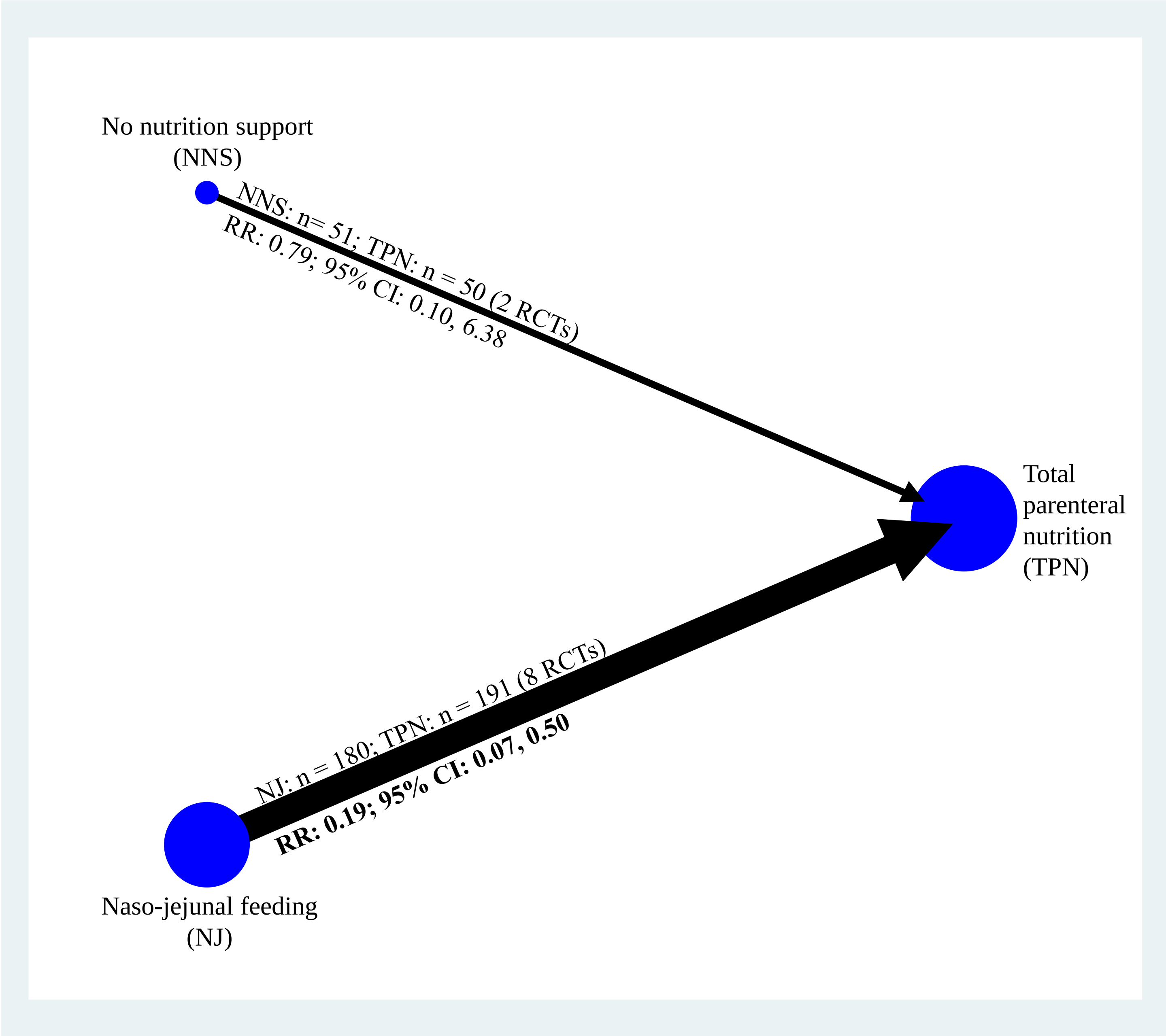

NJ, naso-jejeunal; NNS, no nutrition support; TPN, total parenteral nutrition.

Appendix F2

Ranking probability and SUCRA of line infection

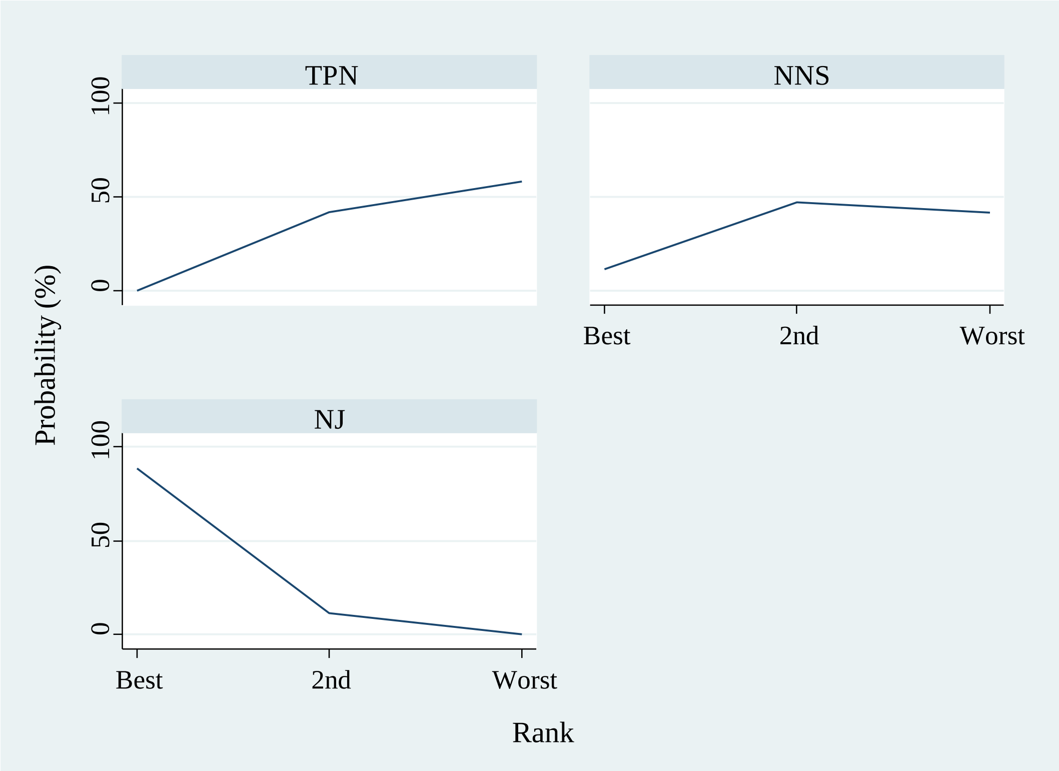

. sucra prob*, labels(TPN NNS NJ)

Treatm~t	SUCRA	PrBest	MeanRank
TPN	20.9	0.0	2.6
NNS	34.9	11.4	2.3
NJ	94.3	88.6	1.1

NJ, naso-jejeunal; NNS, no nutrition support; TPN, total parenteral nutrition.

Appendix F3

Small study bias in network meta-analysis of line infection

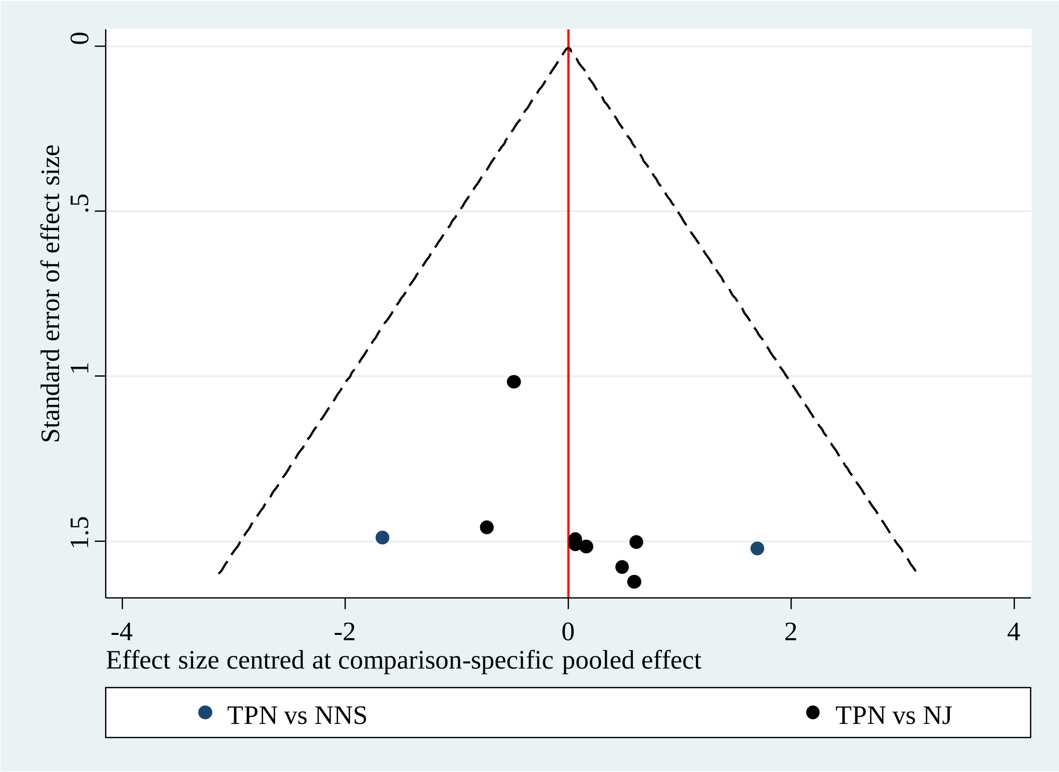

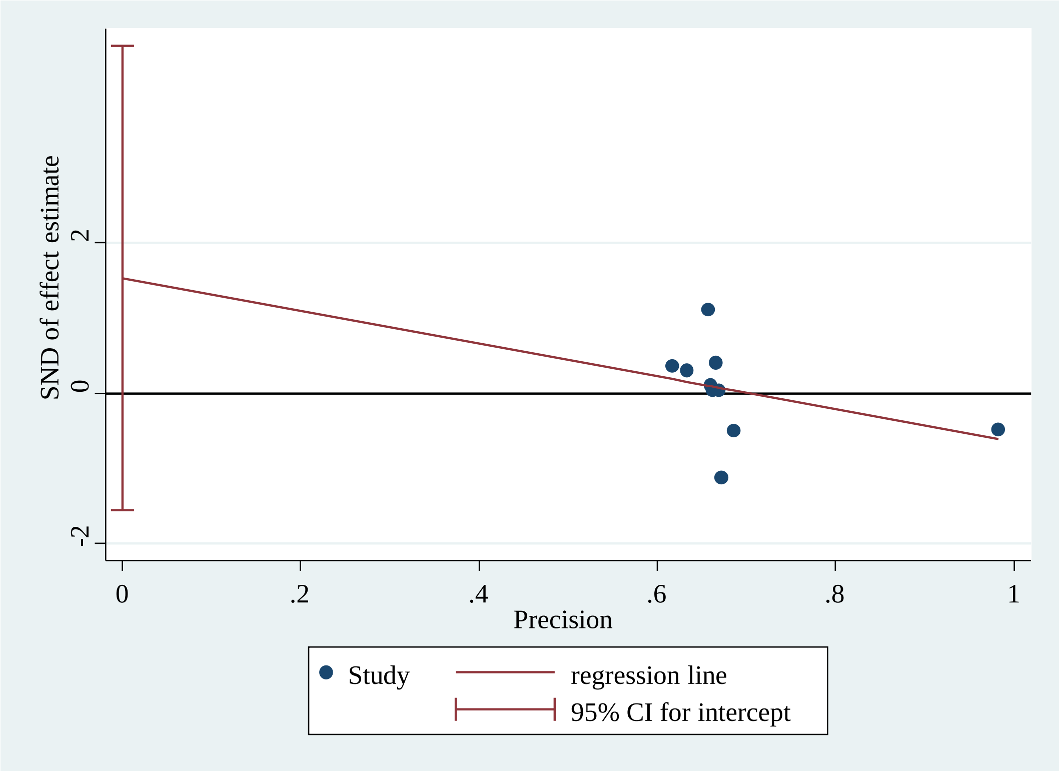

NJ, naso-jejeunal; NNS, no nutrition support; TPN, total parenteral nutrition.

Appendix G1 to G3
Outcomes of pneumonia

Appendix 22
Network geometry of pneumonia

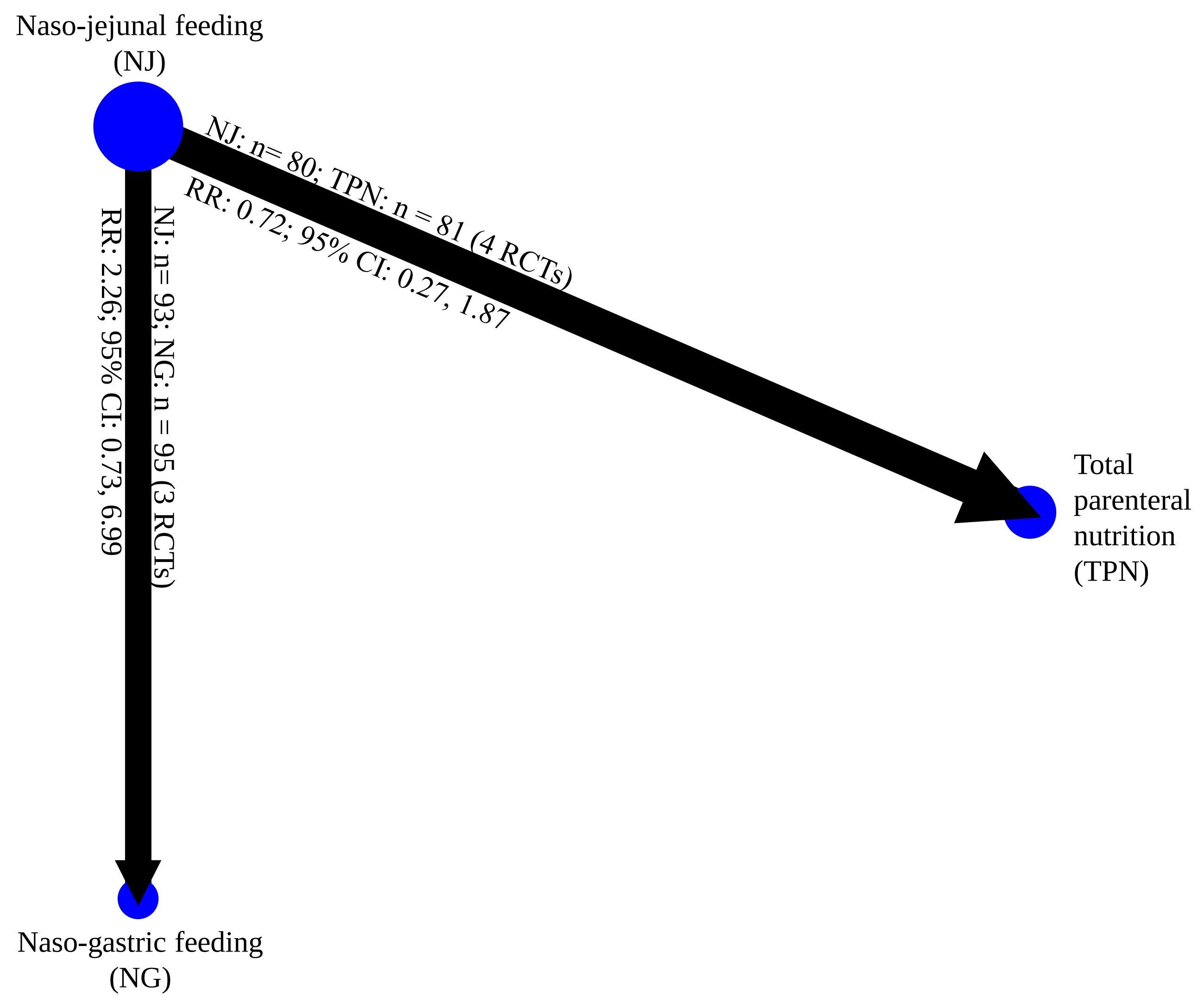

NG, naso-gastric; NJ, naso-jejeunal; TPN, total parenteral nutrition.

Appendix 23
Ranking probability and SUCRA of pneumonia

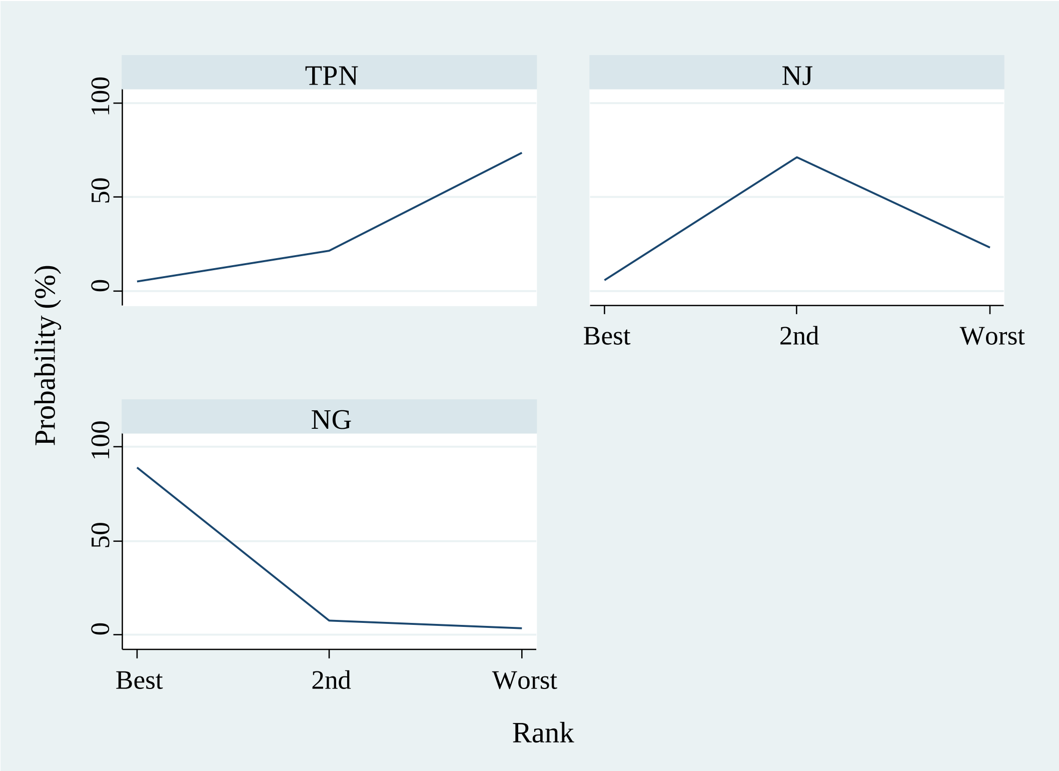


```
. sucra prob*, labels(TPN NJ NG)

  +--------------------------------------+
  | Treatm~t | SUCRA | PrBest | MeanRank |
  |----------+-------+--------+----------|
  |      TPN |  15.8 |    5.1 |      2.7 |
  |       NJ |  41.4 |    5.8 |      2.2 |
  |       NG |  92.9 |   89.1 |      1.1 |
  +--------------------------------------+
```

NG, naso-gastric; NJ, naso-jejeunal; TPN, total parenteral nutrition.

Appendix 24

Small study bias in network meta-analysis of pneumonia

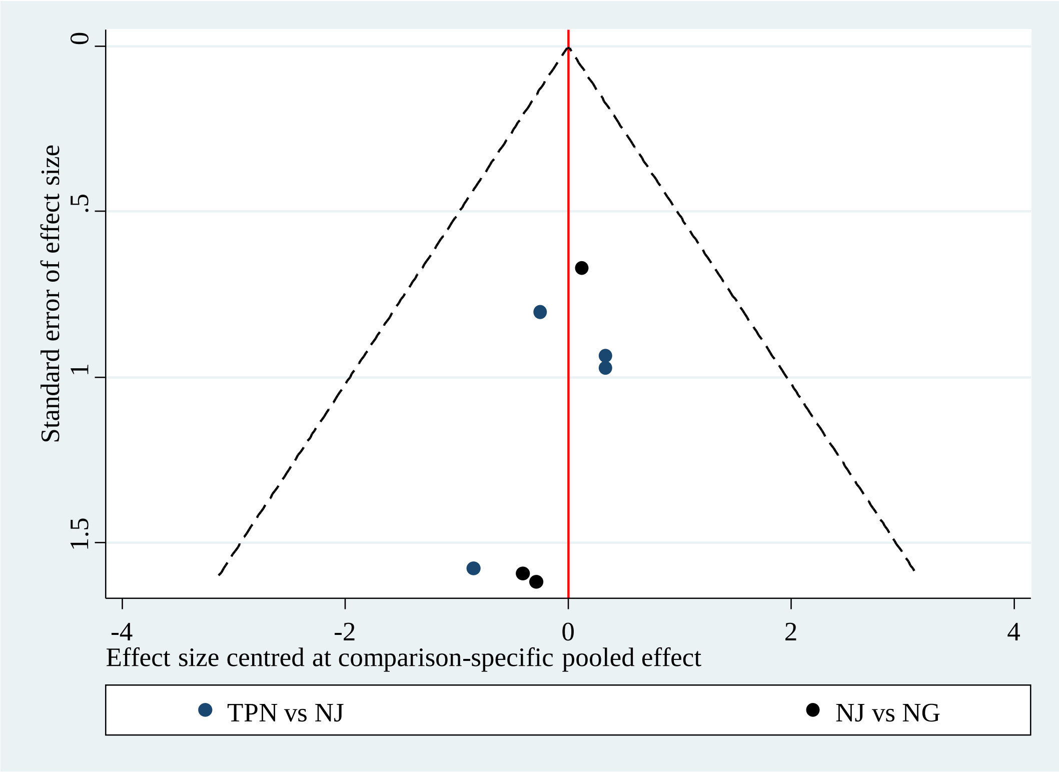

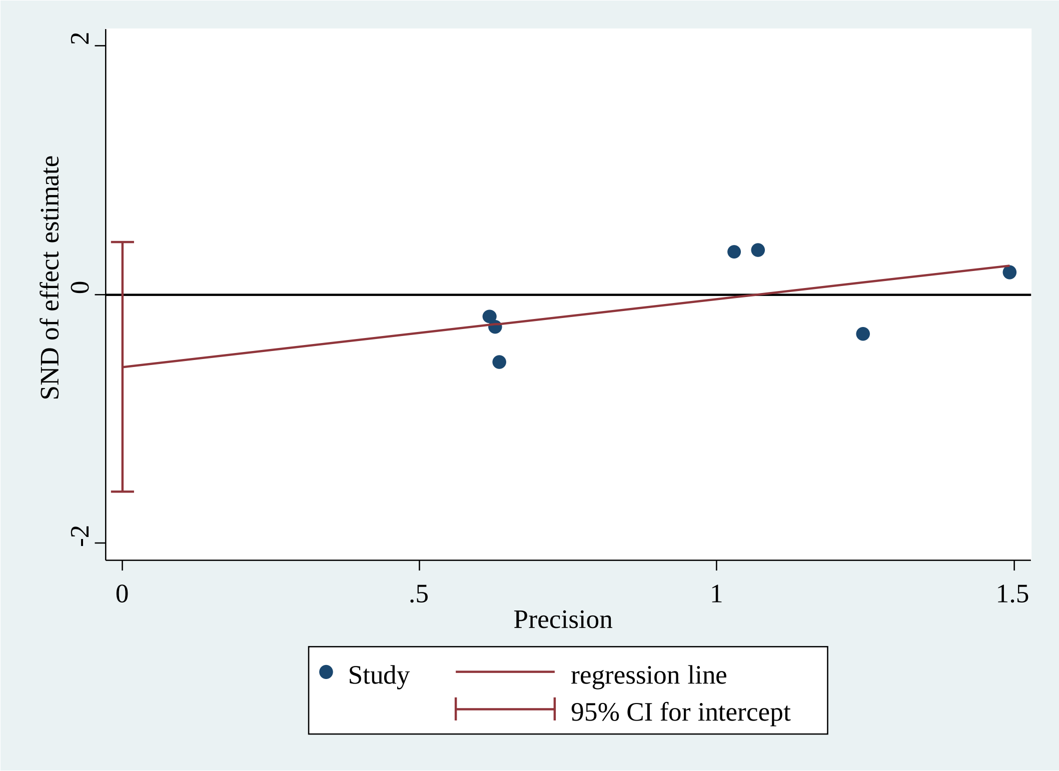

NG, naso-gastric; NJ, naso-jejeunal; TPN, total parenteral nutrition.

Appendix H1 to H2
Outcomes of urinary tract infection

Appendix H1

Pairwise meta-analysis of urinary tract infection

Urinary tract infection

Study name	Risk ratio	Lower limit	Upper limit	Z-Value	p-Value
Casas 2007	3.000	0.135	66.526	0.695	0.487
Gupta 2003	3.333	0.155	71.900	0.768	0.442
Kalfarentzos 1997	0.556	0.055	5.622	-0.498	0.619
McClave 1997	0.500	0.050	4.978	-0.591	0.554
Petrov 2006	0.486	0.095	2.480	-0.868	0.385
	0.755	0.272	2.092	-0.541	0.589

Statistics for each study

Risk ratio and 95% CI

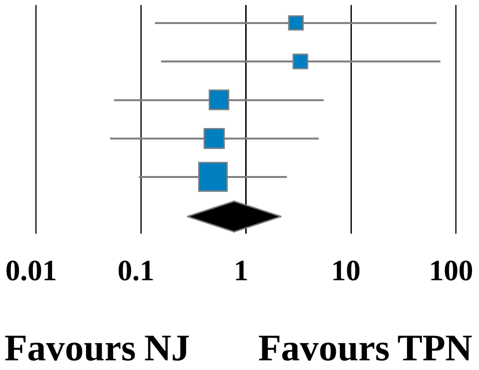

I-square = 0%; Tau-square = 0.00; Q = 2.13; df = 4.

NG, naso-gastric; NJ, naso-jejeunal.

Appendix H2

Small study bias in pairwise meta-analysis of urinary tract infection

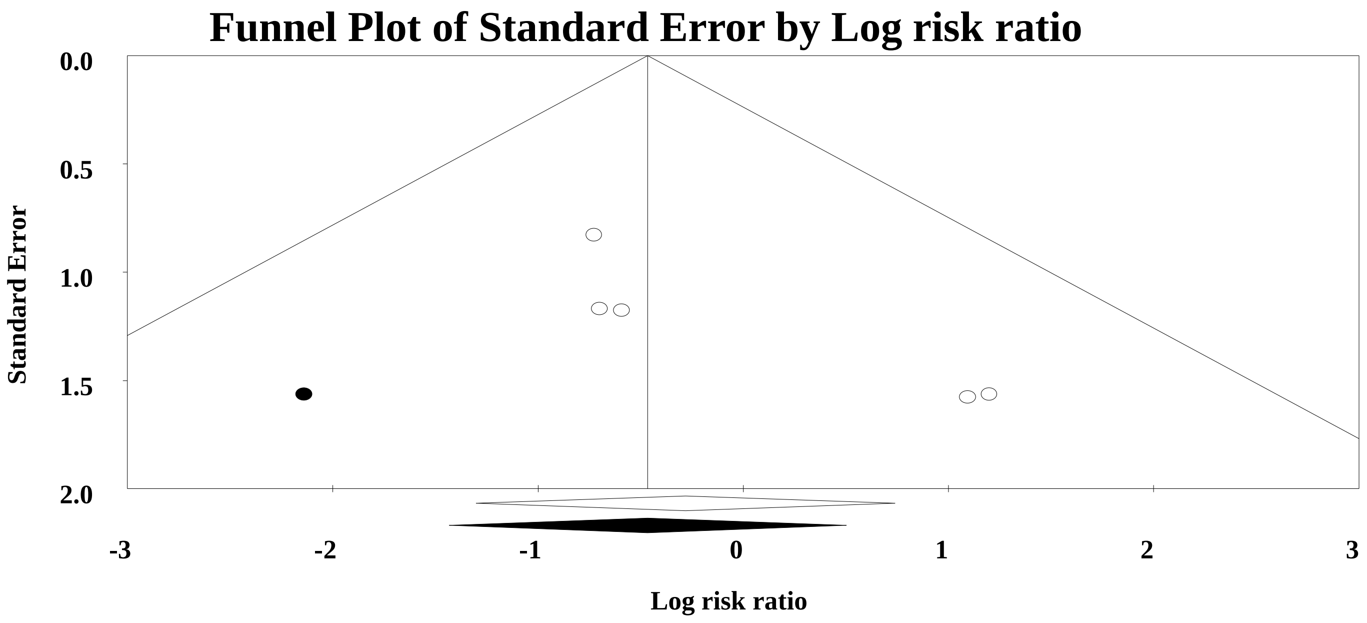

Begg and Mazumdar rank correlation

**Kendall's S statistic (P-Q)**	8.00000

Kendall's tau without continuity correction

Tau	0.80000
z-value for tau	1.95959
P-value (1-tailed)	0.02502
P-value (2-tailed)	0.05004

Kendall's tau with continuity correction

Tau	0.70000
z-value for tau	1.71464
P-value (1-tailed)	0.04321
P-value (2-tailed)	0.08641

Egger's regression intercept

Intercept	2.33613
Standard error	0.79012
95% lower limit (2-tailed)	-0.17838
95% upper limit (2-tailed)	4.85063
t-value	2.95669
df	3.00000
P-value (1-tailed)	0.02985
P-value (2-tailed)	0.05970

Appendix I1 to I4
Outcomes of other type infection

Appendix I1

Network geometry of other type infection

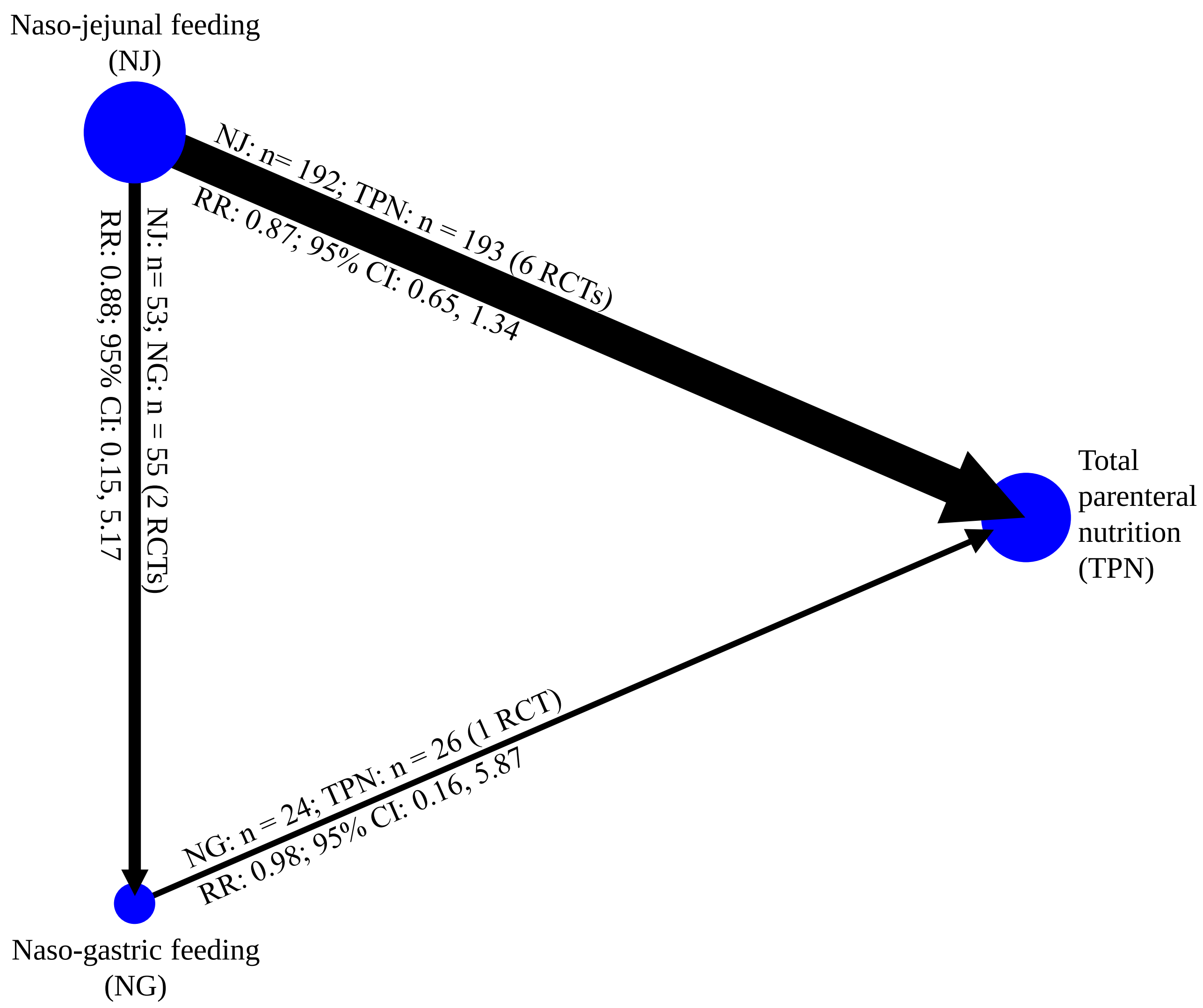

NG, naso-gastric; NJ, naso-jejeunal; TPN, total parenteral nutrition.

Appendix I2

Ranking probability and SUCRA of other type infection

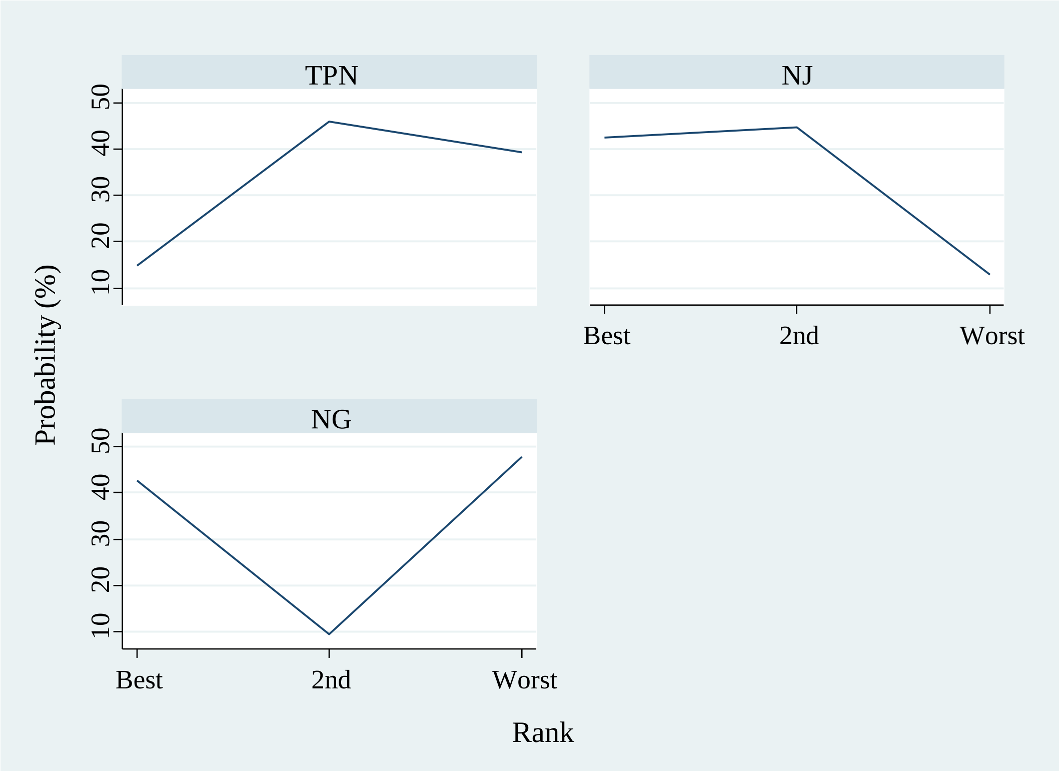


```
. sucra prob*, labels(TPN NJ NG)

  +--------------------------------------+
  | Treatm~t | SUCRA | PrBest | MeanRank |
  |----------+-------+--------+----------|
  |      TPN |  37.8 |   14.8 |      2.2 |
  |       NJ |  64.8 |   42.5 |      1.7 |
  |       NG |  47.4 |   42.7 |      2.1 |
  +--------------------------------------+
```

NG, naso-gastric; NJ, naso-jejeunal; TPN, total parenteral nutrition.

Appendix I3

Inconsistency test for network meta-analysis of other type infection

```
. network meta i, luades

initial:       log likelihood = -21.613544
rescale:       log likelihood = -20.632469
rescale eq:    log likelihood = -20.225308
Iteration 0:   log likelihood = -20.225308
Iteration 1:   log likelihood = -20.171264
Iteration 2:   log likelihood = -20.170227
Iteration 3:   log likelihood = -20.170226

Multivariate meta-analysis
Method = reml                                   Number of dimensions    =     2
Restricted log likelihood = -20.170226          Number of observations  =     9
------------------------------------------------------------------------------
             |      Coef.   Std. Err.      z    P>|z|     [95% Conf. Interval]
-------------+----------------------------------------------------------------
_y_B         |
       _cons |  -.1781658   .2338973    -0.76   0.446    -.6365961    .2802644
-------------+----------------------------------------------------------------
_y_C         |
      groupB |  -2.595667   1.921182    -1.35   0.177    -6.361115    1.169781
       _cons |   1.686399   1.555096     1.08   0.278    -1.361532     4.73433
------------------------------------------------------------------------------

Estimated between-studies SDs and correlation matrix:
            SD        _y_B        _y_C
_y_B  .30880289           1           .
_y_C  .30880289          .5           1

Testing for inconsistency:
 ( 1)  [_y_C]groupB = 0

           chi2(  1) =    1.83
         Prob > chi2 =    0.1767
```

Appendix I4

Small study bias in network meta-analysis of other type infection

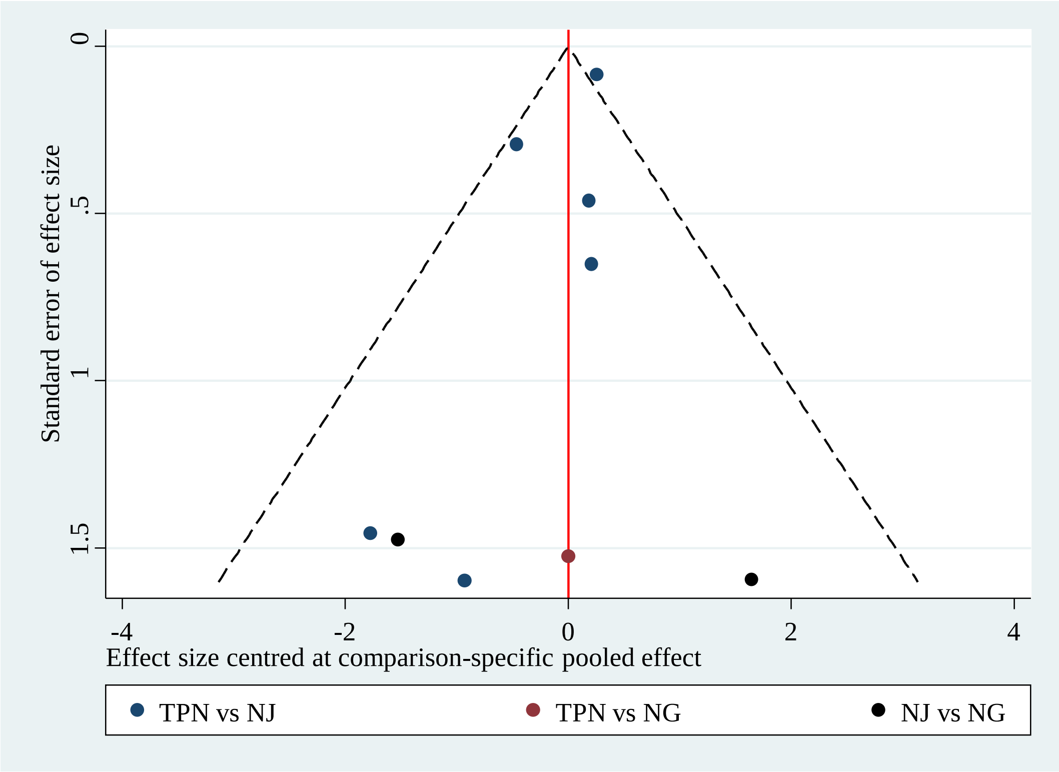

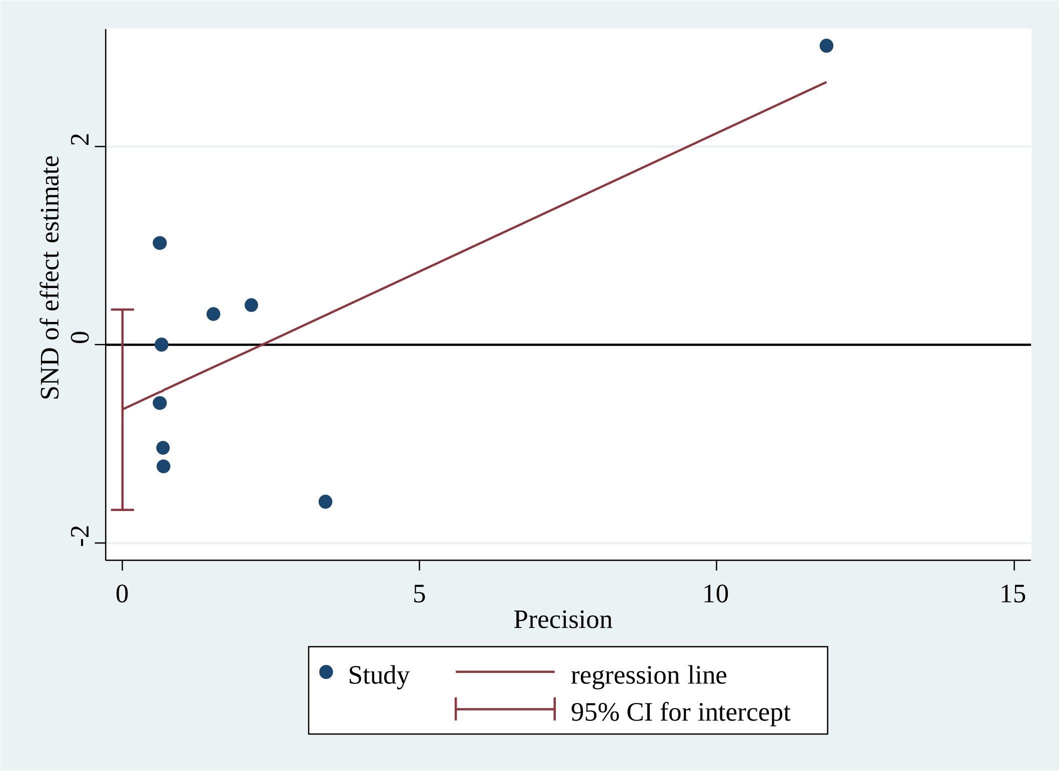

NG, naso-gastric; NJ, naso-jejeunal; TPN, total parenteral nutrition.
