# Supplementary material for: Infection rate among nutritional therapies for acute pancreatitis: A systematic review with network meta-analysis of randomized controlled trials
Source: PLoS One. 2019 Jul 10;14(7):e0219151. doi: 10.1371/journal.pone.0219151 (PMC6620007; doi:10.1371/journal.pone.0219151)
Supplement: S1 Table — (PDF) [file pone.0219151.s003.pdf]

S1 Table

Database and search strategy

Supplementary Table 1 | Database and search strategy

Database	Syntax		Returns
Cochrane	#1	(acute pancreatitis)	1873
	#2	(naso jejunal feeding OR nj OR nasojejunal feeding)	9986
	#3	(nose feeding OR ng OR naso gastric feeding OR nasogastric feeding)	22925
	#4	(nil by mouth OR nbm OR nothing by mouth OR nothing by os OR nothing per os OR nil per os OR npo)	503
	#5	(tpn OR Total parenteral nutrition)	2396
	#6	(oral feeding)	2580
	#7	#1 AND #2 AND #3	29*
	#8	#1 AND #2 AND #4	4*
	#9	#1 AND #2 AND #5	13*
	#10	#1 AND #2 AND #6	15*
	#11	#1 AND #3 AND #4	10*
	#12	#1 AND #3 AND #5	12*
	#13	#1 AND #3 AND #6	23*
	#14	#1 AND #4 AND #5	4*
	#15	#1 AND #4 AND #6	8*
	#16	#1 AND #5 AND #6	14*

* exported and imported to EndNote (Total n = 132).

S1 Table

Database and search strategy

View fewer lines Print

#1	(acute pancreatitis)	S	1873
#2	(naso jejunal feeding OR nj OR nasojejunal feeding)	S	9986
#3	(nose feeding OR ng OR naso gastric feeding OR nasogastric feeding)	S	22925
#4	(nil by mouth OR nbm OR nothing by mouth OR nothing by os OR nothing per os OR nil per os OR npo)	S	503
#5	(tpn OR Total parenteral nutrition)	S	2396
#6	(oral feeding)	S	2580
#7	(#1 AND #2 AND #3)	S	29
#8	(#1 AND #2 AND #4)	S	4
#9	(#1 AND #2 AND #5)	S	13
#10	(#1 AND #2 AND #6)	S	15
#11	(#1 AND #3 AND #4)	S	10
#12	(#1 AND #3 AND #5)	S	12
#13	(#1 AND #3 AND #6)	S	23
#14	(#1 AND #4 AND #5)	S	4
#15	(#1 AND #4 AND #6)	S	8
#16	(#1 AND #5 AND #6)	S	14

S1 Table

Database and search strategy

Database		Syntax	Returns
Embase	#1	'acute pancreatitis'/exp OR 'acute pancreatitis' OR 'pancreatitis acuta'	37829
	#2	'naso jejunal feeding' OR nj OR 'nasojejunal feeding'	228310
	#3	'nose feeding'/exp OR 'feeding, nose' OR 'naso-gastric feeding' OR 'naso-gastric tube feeding' OR 'nasogastric tube feeding' OR 'nose feeding' OR 'tube feeding, nasogastric' OR ng OR 'naso gastric feeding' OR 'nasogastric feeding'	358246
	#4	'nil by mouth' OR nbm OR 'nothing by mouth' OR 'nothing by os' OR 'nothing per os' OR 'nil per os' OR npo	6815
	#5	tpn OR 'total parenteral nutrition'/exp OR 'parenteral nutrition, home total' OR 'parenteral nutrition, total' OR 'total parenteral feeding' OR 'total parenteral nutrition'	20270
	#6	'oral feeding'/exp OR 'oral feeding'	4146
	#7	#1 AND #2 AND #3	60*
	#8	#1 AND #2 AND #4	3*
	#9	#1 AND #2 AND #5	26*
	#10	#1 AND #2 AND #6	4*
	#11	#1 AND #3 AND #4	15*
	#12	#1 AND #3 AND #5	37*
	#13	#1 AND #3 AND #6	12*
	#14	#1 AND #4 AND #5	15*
	#15	#1 AND #4 AND #6	10*
	#16	#1 AND #5 AND #6	9*

* exported and imported to EndNote (Total n = 191).

S1 Table

Database and search strategy

Embase®

Search | Emtree | Journals | Results | My tools | Register | Login | (1)

Results Filters

\+ Expand — Collapse all Apply

- Sources
- Drugs
- Diseases
- Devices
- Floating Subheadings
- Age
- Gender
- Study types
- Publication types
- Journal titles
- Publication years
- Authors

History Save | Delete | Print view | Export | Email Combine using And Or Collapse

#16	#1 AND #5 AND #6	9
#15	#1 AND #4 AND #6	10
#14	#1 AND #4 AND #5	15
#13	#1 AND #3 AND #6	12
#12	#1 AND #3 AND #5	37
#11	#1 AND #3 AND #4	15
#10	#1 AND #2 AND #6	4
#9	#1 AND #2 AND #5	26
#8	#1 AND #2 AND #4	3
#7	#1 AND #2 AND #3	60
#6	**'oral feeding'**/exp OR **'oral feeding'**	4,146
#5	**tpn** OR **'total parenteral nutrition'**/exp OR **'parenteral nutrition, home total'** OR **'parenteral nutrition, total'** OR **'total parenteral feeding'** OR **'total parenteral nutrition'**	20,270
#4	**'nil by mouth'** OR **nbm** OR **'nothing by mouth'** OR **'nothing by os'** OR **'nothing per os'** OR **'nil per os'** OR **npo**	6,815
#3	**'nose feeding'**/exp OR **'feeding, nose'** OR **'naso-gastric feeding'** OR **'naso-gastric tube feeding'** OR **'nasogastric tube feeding'** OR **'nose feeding'** OR **'tube feeding, nasogastric'** OR **ng** OR **'naso gastric feeding'** OR **'nasogastric feeding'**	358,246
#2	**'naso jejunal feeding'** OR **nj** OR **'nasojejunal feeding'**	228,310
#1	**'acute pancreatitis'**/exp OR **'acute pancreatitis'** OR **'pancreatitis acuta'**/exp OR **'pancreatitis acuta'**	37,829

S1 Table

Database and search strategy

Database		Syntax	Returns
PubMed	#1	acute pancreatitis	30761
	#2	((naso jejunal feeding) OR nj) OR nasojejunal feeding	73687
	#3	(((nose feeding) OR ng) OR naso gastric feeding) OR nasogastric feeding	253637
	#4	((((((nil by mouth) OR nbm) OR nothing by mouth) OR nothing by os) OR nothing per os) OR nil per os) OR npo	3004
	#5	(tpn) OR Total parenteral nutrition	16581
	#6	oral feeding	20092
	#7	((acute pancreatitis) AND (((naso jejunal feeding) OR nj) OR nasojejunal feeding)) AND ((((nose feeding) OR ng) OR naso gastric feeding) OR nasogastric feeding)	29*
	#8	((acute pancreatitis) AND (((naso jejunal feeding) OR nj) OR nasojejunal feeding)) AND (((((((nil by mouth) OR nbm) OR nothing by mouth) OR nothing by os) OR nothing per os) OR nil per os) OR npo)	3*
	#9	((acute pancreatitis) AND (((naso jejunal feeding) OR nj) OR nasojejunal feeding)) AND ((tpn) OR Total parenteral nutrition)	20*
	#10	((acute pancreatitis) AND (((naso jejunal feeding) OR nj) OR nasojejunal feeding)) AND oral feeding	15*
	#11	((acute pancreatitis) AND ((((nose feeding) OR ng) OR naso gastric feeding) OR nasogastric feeding)) AND (((((((nil by mouth) OR nbm) OR nothing by mouth) OR nothing by os) OR nothing per os) OR nil per os) OR npo)	7*
	#12	((acute pancreatitis) AND ((((nose feeding) OR ng) OR naso gastric feeding) OR nasogastric feeding)) AND ((tpn) OR Total parenteral nutrition)	12*
	#13	((acute pancreatitis) AND ((((nose feeding) OR ng) OR naso gastric feeding) OR nasogastric feeding)) AND oral feeding	16*
	#14	((acute pancreatitis) AND (((((((nil by mouth) OR nbm) OR nothing by mouth) OR nothing by os) OR nothing per os) OR nil per os) OR npo)) AND ((tpn) OR Total parenteral nutrition)	12*
	#15	((acute pancreatitis) AND (((((((nil by mouth) OR nbm) OR nothing by mouth) OR nothing by os) OR nothing per os) OR nil per os) OR npo)) AND oral feeding	5*
	#16	((acute pancreatitis) AND ((tpn) OR Total parenteral nutrition)) AND oral feeding	14*

* exported and imported to EndNote (Total n = 133).

S1 Table

Database and search strategy

History

Download history Clear history

Search	Add to builder	Query	Items found	Time
#17	Add	Search **((acute pancreatitis) AND ((tpn) OR Total parenteral nutrition)) AND oral feeding**	14	10:30:04
#16	Add	Search **((acute pancreatitis) AND ((((((nil by mouth) OR nbm) OR nothing by mouth) OR nothing by os) OR nothing per os) OR nil per os) OR npo)) AND oral feeding**	5	10:29:52
#15	Add	Search **((acute pancreatitis) AND ((((((nil by mouth) OR nbm) OR nothing by mouth) OR nothing by os) OR nothing per os) OR nil per os) OR npo)) AND ((tpn) OR Total parenteral nutrition)**	12	10:29:35
#14	Add	Search **((acute pancreatitis) AND ((((nose feeding) OR ng) OR naso gastric feeding) OR nasogastric feeding)) AND oral feeding**	16	10:29:13
#13	Add	Search **((acute pancreatitis) AND ((((nose feeding) OR ng) OR naso gastric feeding) OR nasogastric feeding)) AND ((tpn) OR Total parenteral nutrition)**	12	10:29:03
#12	Add	Search **((acute pancreatitis) AND ((((nose feeding) OR ng) OR naso gastric feeding) OR nasogastric feeding)) AND ((((((nil by mouth) OR nbm) OR nothing by mouth) OR nothing by os) OR nothing per os) OR nil per os) OR npo)**	7	10:28:53
#11	Add	Search **((acute pancreatitis) AND (((naso jejunal feeding) OR nj) OR nasojejunal feeding)) AND oral feeding**	15	10:28:42
#10	Add	Search **((acute pancreatitis) AND (((naso jejunal feeding) OR nj) OR nasojejunal feeding)) AND ((tpn) OR Total parenteral nutrition)**	20	10:28:26
#9	Add	Search **((acute pancreatitis) AND (((naso jejunal feeding) OR nj) OR nasojejunal feeding)) AND ((((((nil by mouth) OR nbm) OR nothing by mouth) OR nothing by os) OR nothing per os) OR nil per os) OR npo)**	3	10:27:08
#8	Add	Search **((acute pancreatitis) AND (((naso jejunal feeding) OR nj) OR nasojejunal feeding)) AND ((((nose feeding) OR ng) OR naso gastric feeding) OR nasogastric feeding)**	29	10:26:59
#7	Add	Search **oral feeding**	20691	10:26:50
#6	Add	Search **(tpn) OR Total parenteral nutrition**	16832	10:26:42
#5	Add	Search **((((((nil by mouth) OR nbm) OR nothing by mouth) OR nothing by os) OR nothing per os) OR nil per os) OR npo**	3189	10:26:30
#4	Add	Search **(((nose feeding) OR ng) OR naso gastric feeding) OR nasogastric feeding**	260510	10:26:14
#3	Add	Search **((naso jejunal feeding) OR nj) OR nasojejunal feeding**	78137	10:25:17
#2	Add	Search **acute pancreatitis**	31515	10:25:04

S1 Table

Database and search strategy

Database		Syntax	Returns
Web of Science (core collection)	#1	(acute pancreatitis)	20877
	#2	(naso jejunal feeding OR nj OR nasojejunal feeding)	9071
	#3	(nose feeding OR ng OR naso gastric feeding OR nasogastric feeding)	195235
	#4	(nil by mouth OR nbm OR nothing by mouth OR nothing by os OR nothing per os OR nil per os OR npo)	2366
	#5	(tpn OR Total parenteral nutrition)	104457
	#6	(oral feeding)	18641
	#7	(acute pancreatitis) AND (naso jejunal feeding OR nj OR nasojejunal feeding) AND (nose feeding OR ng OR naso gastric feeding OR nasogastric feeding)	29*
	#8	(acute pancreatitis) AND (naso jejunal feeding OR nj OR nasojejunal feeding) AND (nil by mouth OR nbm OR nothing by mouth OR nothing by os OR nothing per os OR nil per os OR npo)	3*
	#9	(acute pancreatitis) AND (naso jejunal feeding OR nj OR nasojejunal feeding) AND (tpn OR Total parenteral nutrition)	25*
	#10	(acute pancreatitis) AND (naso jejunal feeding OR nj OR nasojejunal feeding) AND (oral feeding)	13*
	#11	(acute pancreatitis) AND (nose feeding OR ng OR naso gastric feeding OR nasogastric feeding) AND (nil by mouth OR nbm OR nothing by mouth OR nothing by os OR nothing per os OR nil per os OR npo)	7*
	#12	(acute pancreatitis) AND (nose feeding OR ng OR naso gastric feeding OR nasogastric feeding) AND (tpn OR Total parenteral nutrition)	29*
	#13	(acute pancreatitis) AND (nose feeding OR ng OR naso gastric feeding OR nasogastric feeding) AND (oral feeding)	12*
	#14	(acute pancreatitis) AND (nil by mouth OR nbm OR nothing by mouth OR nothing by os OR nothing per os OR nil per os OR npo) AND (tpn OR Total parenteral nutrition)	14*
	#15	(acute pancreatitis) AND (nil by mouth OR nbm OR nothing by mouth OR nothing by os OR nothing per os OR nil per os OR npo) AND (oral feeding)	4*
	#16	(acute pancreatitis) AND (tpn OR Total parenteral nutrition) AND (oral feeding)	25*

* exported and imported to EndNote (Total n = 161).
