# Supplementary material for: Infection rate among nutritional therapies for acute pancreatitis: A systematic review with network meta-analysis of randomized controlled trials
Source: PLoS One. 2019 Jul 10;14(7):e0219151. doi: 10.1371/journal.pone.0219151 (PMC6620007; doi:10.1371/journal.pone.0219151)
Supplement: S2 Table — (PDF) [file pone.0219151.s004.pdf]

S2 Table

Risk of bias of the included RCTs

Study	Quality Judgment	Risk bias 1	2	3	4	5	6	7
Abou-Assi et al., 2002	Minor considered	?[8]	?	-[9]	-	+[10]	+	+
Casas et al., 2007	Minor considered	+	+	-	-	+	+	?
Doley et al., 2009	Major considered	-	-	-	-	+	+	+
Du et al.,2015	Major considered	?	?	-	-	+	+	?
Eckerwall et al., 2006	Minor considered	+	+	-	-	+	+	?
Entock et al., 2005	Minor considered	+	+	-	-	+	+	?
Gupta et al., 2003	Minor considered	+	+	–	–	+	+	?
He et al., 2004	Major considered	?	?	-	-	+	+	?
Kalfarentzos et al., 1997	Minor considered	+	+	-	-	+	+	?
Kumar et al., 2006	Minor considered	+	+	-	-	+	+	?
McClave et al., 1997	Major considered	?	?	-	-	+	+	?
Louie et al., 2005	Minor considered	+	+	-	-	+	+	+
MIMOSA trial	Minor considered	+	+	-	-	+	+	+
Olah et al., 2002	Major considered	-	-	-	-	+	+	?
Petrov et al., 2006	Minor considered	+	+	-	-	+	+	?
Powell et al., 2000	Minor considered	?	?	-	-	+	+	+
Sax et al., 1987	Major considered	?	?	-	-	+	+	?
Singh et al., 2012	Minor considered	+	+	-	+	+	+	+
Stimac et al., 2016	Minor considered	+	+	-	-	+	+	+
Wang et al., 2013	Major considered	?	?	-	-	+	+	?
Wu et al., 2010	Major considered	?	?	-	-	+	+	?
Zhang et al., 2011	Major considered	?	?	-	-	+	+	?

1 sequence generation; 2 allocation concealment; 3 blinding of participants and personnel; 4 blinding of outcome assessment; 5 incomplete outcome data; 6 selective reporting; 7 other bias. [8] Yellow with ?, risk unclear; [9] Red with -, high risk; [10] Green with +, low risk.
