# Supplementary material for: Infection rate among nutritional therapies for acute pancreatitis: A systematic review with network meta-analysis of randomized controlled trials
Source: PLoS One. 2019 Jul 10;14(7):e0219151. doi: 10.1371/journal.pone.0219151 (PMC6620007; doi:10.1371/journal.pone.0219151)
Supplement: S3 Table — (PDF) [file pone.0219151.s005.pdf]

S3 Table

Further information of the included RCTs

Study	Recruit year	Year of publication	Country	Therapy (Therapy1 / Therapy2)	Patient (Therapy1 / Therapy2)	Age (Therapy1 / Therapy2)
Abou-Assi, 2002	2000/1-12	2002	North America	NJ/TPN	26/27	48(SD:3)/ 50(SD:3)
Casas, 2007	NR	2007	Spain	NJ/TPN	11/11	61.2(SD:10.7)/ 55.6(SD:15.3)
Doley, 2009	2006/7~2007/12	2009	India	NJ/TPN	25/25	38.4(SD:13.8)/ 41.1(SD:11.3)
Du, 2015	2009/3~2013/12	2015	China	NG/NJ	40/40	41(25-60)/ 43(23-65)
Eckerwall, 2006	2002/1~2004/12	2006	Sweden	NG/TPN	24/26	71(58-80)/ 68(60-80)
Entock, 2005	1997/10~2000/7	2005	Scotland	NG/NJ	27/22	63(47-74)/ 58(48-64)
Gupta, 2003	1996/11~1998/4	2003	United Kingdom	NJ/TPN	11/10	65(56-89)/ 57(38-86)
He, 2004	NR	2004	China	TPN/NNS	22/25	40.2±7.8/ 39.6±5.2
Kalfarentzos, 1997	1990/7~1995/12	1997	Greece	NJ/TPN	18/20	63(SD:10.7)/ 67.2(SD:8.9)
Kumar, 2006	2002/9~2003/12	2006	India	NG/NJ	16/14	43.25(SD:12.76)/ 35.57(SD:12.53)
McClave, 1997	NR	1997	North America	NJ/TPN	16/16	47.64(SEM:4)/ 45.1(SEM:4.2)
Louie, 2005	1999/7/15~2001/12/15	2005	Canada	NJ/TPN	10/18	65.3(SD:18.3)/ 59(SD:15.3)
MIMOSA trial	2010/5~2011/4	2015	New Zealand	NG/NNS	17/18	41(34-60)/ 55(36-70)
Olah, 2002	1995/1/1~1996/5/31	2002	Hungary	NJ/TPN	41/48	47.2/43.8
Petrov, 2006	2002/3~2004/12	2006	Russia	NJ/TPN	35/35	51(IQR:42-67)/ 52(IQR:41-70)
Powell, 2000	1996/12/16~1998/7/1	2000	Scotland	NJ/NNS	13/14	64(26-76)/ 52(23-78)
Sax, 1987	1984/7/1~1985/12/31	1987	America	TPN/NNS	28/26	39.8±2/ 39.8±3
Singh, 2012	2005/1~2007/12	2012	India	NG/NJ	39/39	39.1(SD:16.7)/ 39.7(SD:12.3)
Stimac, 2016	2007/5~2012/2	2016	Croatia	NJ/NNS	107/107	69(IQR:28-88)/ 72(IQR:26-90)
Wang, 2013	2006/1~2011/12	2013	China	NJ/TPN	61/60	43.145±13.75/ 41.7±11.4
Wu, 2010	2003/11~2007/12	2010	China	NJ/TPN	53/54	52±12.1/ 54±11.2
Zhang, 2011	2006/1~2009/10	2011	China	NJ/TPN	42/42	47.4±9.9/ 48.6±10.5

S3 Table

Further information of the included RCTs

Study	Inclusion criteria	Severity	
		Therapy 1	Therapy 2
Abou-Assi, 2002	NR	Ranson's score: 3.1(0.5)	Ranson's score: 2.5(0.4)
Casas, 2007	APACHEII>8, CRP>150, Balthazar D or E in abd. CT	NR	NR
Doley, 2009	Atlanta criteria:<br>Contrast enhanced CT evidence of pancreatic necrosis<br>CTSI≥7	CTSI:8.84±1.07	CTSI:8.72±1.14
Du, 2015	Severity according to 2009<br>Chinese acute pancreatitis criteria	APACHEII:<br>17(13~27)	APACHEII:<br>16(12~28)
Eckerwall, 2006	APACHEII ≥8 or CRP≥150mg/L<br>And/or peri-pancreatic liquid shown on CT	APACHEII:10, CRP:128	APACHEII:9, CRP:113
Entock, 2005	APACHEII≥6, GPS≥3, CRP>150	NR	NR
Gupta, 2003	APACHE≥6	APACHEII: 8(6-12)	APACHEII: 10(7-14)
He, 2004	NR	NR	NR
Kalfarentzos, 1997	APACHEII≥8 or CRP>120mg/L within 48h of admission Grade D or E by CT according to the Balthazar criteria	APACHEII:12.7 (SD:2.6)<br>Imrie:4.2(SD:0.9), CRP:290(157-420)	APACHEII:11.8 (SD:1.9),<br>Imrie:4.6(SD:1.1), CRP:335(140-513)
Kumar, 2006	APACHEII≥8<br>Or CTSI≥7	APACHE II: 10.50±3.78, CTSI:6.07±2.87	APACHEII:9.64±4.99, CTSI:6.86±2.54
McClave, 1997	Ranson:1.3±0.35<br>(range:0-5)	APACHEIII: 17.5±4.1, Ranson:1.3±0.35,<br>MOF score: 1.3±0.45	APACHEIII: 22.4 ± 5.0, Ranson: 1.3 ± 0.35,<br>MOF score: 1.1 ± 0.49
Louie, 2005	Ranson≥3	Ranson:4.7(1.3), APACHEII:11.8(8.3),<br>Balthazar:3.4(1.3)<br>Has rule out severe and initial use oral feeding	Ranson:5.0(1.8), APACHEII:12.7(5.5),<br>Balthazar:4.2(0.8)
MIMOSA trial	Rule out severe<br>Severe: presence of organ failure and/or pancreatic infection at the time of randomization	APACHEII 6(2-9), BISAP:0(0-1)<br>CRP:44(6-167), Glasgow score: 1(0-2)	APACHEII 6(3-11), BISAP:0(0-2)<br>CRP: 66(16-145), Glasgow score:2(0-2)
Olah, 2002		Imrie score≥3; CRP>150mg/L;<br>APACHEII>6	Imrie score≥3; CRP>150mg/L;<br>APACHEII>6
Petrov, 2006	APACHE≥8 or CRP≥150mg/l	APACHEII: 12(10-14), CRP:195(164-216)	APACHEII: 12.5(11-16), CRP:210(177-246)
Powell, 2000	Glasgow score>3, APACHEII>7	APACHEII: 10(7-20), Glasgow:4(1-5),<br>Helsinki:4(1-6), Balthazar:3(0-8)	APACHEII: 12(7-32), Glasgow:4(1-6),<br>Helsinki:4(0-7), Balthazar:2(0-8)
Sax, 1987	NR	Ranson score: 1.1±0.2	Ranson score: 0.92±0.17
Singh, 2012	APACHE≥8; CTSI≥7; Presence of ≥1 organ failure defined by Atlanta classification	APACHE:8.5(2-19), CTSI:5.76(2.51)	APACHEII:8(2-24), CTSI:5.96(2.57)
Stimac, 2016	APACHEII≥6	APACHEII:9.84(3.26), Ranson:2.98(1.69),<br>CTSI:2.64(2.73)	APACHEII:9.74(4.06), Ranson:2.83(1.81),<br>CTSI:2.84(2.78)
Wang, 2013	Use Atlanta criteria	APACHE:13.27±2.86, Ranson:4.98±2.16,<br>CTSI:6.68±2.18,Marshall score: 4.88±1.65	APACHEII:14.63±3.67, Ranson:5.12±1.92,<br>CTSI:6.71±1.79, Marshall score:4.95±1.82
Wu, 2010	Pancreatic necrosis<br>CRP≥ 19.5mg/dL	APACHEII:14±2.1, CRP:211±9.2	APACHE:16±4.4, CRP:218±7.9
Zhang, 2011	APACHE>8, Ranson>3	APACHE:12.8±3.1, Ranson: 3.8±1.3	APACHE:13.1±2.3,<br>Ranson: 3.7±0.9

Supplemental Table 3

Further information of the included RCTs

Study	Symptoms onset (Therapy1 / Therapy2)	Nutrition intervention time (Therapy1 / Therapy2)	Antibiotics use (Therapy1 / Therapy2)	Calorie target (Therapy1 / Therapy2)	Fulfillment of target calorie
Abou-Assi, 2002	NR	Weaning from nutritional support was attempted when abdominal pain and distention had settled and enzyme elevations had consistently decreased toward normal levels over 3 days.	1/9 with line infection	1.5 protein/kg/day and 25-30kcal/kg/day	Enteral: 49% estimated caloric and 42% of protein requirements. Parenteral: 85% of protein and caloric. Route 1<Route 2
Casas, 2007	NR	All patients were submitted to intensive control for at least the first 72 hours after admission. with a maximum of 72 hours, all patients were randomized and distributed to one of the two groups, and the assigned nutrition was immediately started.	Eleven patients, 6 from group I (TPN)and 5 (NJ)from group II, were treated with antibiotics. Two patients from group I and all patients in group II were treated with imipenem, whereas the remaining 4 patients in group I were treated with piperacillin-tazobactam.	1.5-2 g proteins/kg/day and 30-35 kcal/kg/day.	Route1 (20.09±1.83 kcal/kg/day)<br>Route 2 (20.8±1.68 kcal/kg/day)
Doley, 2009	Duration of pancreatitis at admission: 3.52±0.92	Nutritional support was initiated within 72 hours of admission	prophylactic antibiotics (ciprofloxacin/metronidazole or imipenem/cilastatin)	2500-2700kcal/day and 120-130g/day protein	All, in 14 days<br>(route 1=route 2)
Du, 2015	NR	start after admission 3-5 days	FQ+ metronidazole	25-30kal/kg/day	Route 1=Route 2<br>inadequate support with PN
Eckerwall, 2006	Onset of abdominal pain within 48hr	the nutritional support to start within 24 hours from admission.<br>19(14-24)/17(10-24)	Broad-spectrum antibiotic therapy was used according to current recommendations.<br>No significant difference was seen between the groups in the frequency of antibiotic prophylaxis (17 of 25 vs. 18 of 23; *P*>0.30).	Isocaloric between route 1 and route 2 , energy target of 25cal/kg/day,	Achieved in 66% in both group<br>(route 1=route 2)
Entock, 2005	NR	Feeding start from onset of pain 72hr (24-72)	NR	2000 kcal / day	77.8% in NG/76.1% in NJ
Gupta, 2003	NR	Enteral nutritional support was commenced within 6 h of the diagnosis of predicted severe acute pancreatitis being made/ TPN started ASAP after diagnosis	all use antibiotics (cefuroxime 750 mg tds).	36 kcal/kg/day	Route 1 =Route 2<br>(patients who received nutritional support were given similar numbers of calories in each group)
He, 2004	NR	NR	Prophylactic antibiotics use	(104.5 kJ/kg/d)	NR
Kalfarentzos, 1997	Mean duration of symptoms :1.7 days(1.3)/1.8 days(1.5)	NJ placed within the first 48hr after admission began immediately after patient return to ICU/ TPN NR	Prophylactic use of imipenem was started on admission and continued until clinical recovery and restoration of normal C-reactive protein concentrations.	1.5-2g protein/kg/day, 30-35kcal/kg/day	Enteral 24.1 v.s. TPN 24.5 kcal /kg/day<br>route 1=route 2 (non-protein caloric intake)<br>1.43 v.s. 1.45g/kg/day protein intake
Kumar, 2006	Days between disease onset and admission to our hospital<br>7.81±6.50/5.71±4.73	Refeeding started in all patients 48hrs after admission	standard medical treatment in form of antibiotics, fluid and electrolytes, and other support for organ failure was given to the patients as indicated.<br>No detail information.	1800kcal	Route1=Route 2<br>All patients achieved the goal of 1800kcal within 7 days from the start of feeding.
McClave, 1997	NR	nutritional support started within 48hrs	No patient received empiric antibiotics on admission for acute pancreatitis. Any antibiotics given were chosen to treat specific nosocomial infections according to bacterial cultures and sensitivities	25kcal/kg/day, 1.2g protein/kg/day	Route 1(72%)= Route 2(TPN:81%)
Louie, 2005	NR	Days on nutrition:<br>13.1(10.5)/14.6(10.3)<br>days to diet as tolerated:15(10.6)/15.9(10.2)	Antibiotic coverage was provided to 79% of patients in the study	25kcal/kg/day, 1.5g/kg/day protein	Route 1 =Route 2<br>Both groups reached the targeted energy intake at similar times, and the daily average kilojoules were also similar.
MIMOSA trial	Duration of symptom 18hr(6-36)	feeding was started at 22 (16-23) h after hospital admission. / 4 (3-5.5) days after hospital admission in the NPO group	NR	Enteral nutrition was started at a rate of 25 mL/h and increased stepwise until 100 mL/h was reached over 24-48 h.	The volume of enteral feed given to each patient was 2710 (1060- 5000) mL
Olah, 2002	Patients admitted within 24 to 72 h after the onset of symptoms were randomized into the study	Therapy was instituted in every case within 24 h of admission	No use	30kcal/kg/day	NR

Supplemental Table 3

Further information of the included RCTs

Study	Symptoms onset (Therapy1 / Therapy2)	Nutrition intervention time (Therapy1 / Therapy2)	Antibiotics use (Therapy1 / Therapy2)	Calorie target (Therapy1 / Therapy2)	Full-fillment of target calorie
Petrov, 2006	Patients with prognostically severe acute pancreatitis within 72 h of the onset of symptoms were included	Nutritional support commenced within 24 h of enrolment for a minimum of 7 days.	Antibiotic prophylaxis (ofloxacin plus metronidazole), and intravenous fluids.	30 kcal/kg/day and 1.5 g/kg/day of protein	Route 1=Route 2 Acheieve 100% of target energy rate.
Powell, 2000	Duration of symptoms(h): 31(10-72)/27(6-68)	NR	All patients were prescribed IV cefotaxime 2g three times daily and metronidazole 500 mg three times daily for 4 days. After 4 days antibiotics therapy was stopped. Further antibiotic therapy was prescribed according to bacteriological cultures or at the discretion of the treating clinician.	500ml Jevity contains 4g protein, 3.5g fat, 13.1g carbohydrate, and 1.4g dietary fibre, providing 2105 kJ. Not mentioned clearly target	Until the desired caloric intake was reached
Sax, 1987	NR	TPN within 24hr admission based on surgeon to stop	NR	In all cases TPN infusion rates were advanced over 2-3days to the estimated needs as determined by the Harris-Benedict equation.	
Singh, 2012	Days between disease onset and admission to hospital (median,range): 5(1-18)/7(1-48)	Days between disease onset and initiation of feeding, median (range) :10(4-23) /11(3-48)	Antibiotics were prescribed if patients had infected pancreatic necrosis or if there was documented infection at the extrapancreatic sites.	25kcal/kg/day	Route 1 =Route 2 Semi-element enteral formula was used to reach the nutrient goal in 3 to 4 days.
Stimac, 2016	the onset of symptoms consistent with AP within 72 h before admission to the hospital	EN provided within 24 h of admission	Antibiotic prophylaxis with imipenem 500 mg i.v. three times a day was administered to all patients during the first ten days.	25kcal/kg/day, 1.5g/kg of protein	When patient were able to receive 50% of their maintenance energy requirements through fluid diet, the rate of EN was halved. Upon achieving this goal and starting the low-fat diet with no occurrence of pain, EN was stopped.
Wang, 2013	the time interval between onset of typical abdominal symptoms and study inclusion was 48 h	TPN or NJ feeding within the first 7 days after hospitalization were included in this study.	prophylaxis with imipenem for pancreatic infection	TPN: 2g proteins/kg/day, and 35kcal/kg/day NJ: 2g proteins/kg/day,30kcal/kg/day	Route 1 =Route 2
Wu, 2010	NR	NR	received sufficient prophylactic antibiotics (intravenous metronidazol/ciprofloxacin)	TPN: 1.2g protein/kg/day, 105(25.11kcal)-126kJ(30.14kcal)/kg/day NJ: 1.5g protein/kg/day, 105-126kJ/kg/day	Route 1 =route 2
Zhang, 2011	NR	After 72hr of admission	received prophylactic antibiotics	25~35kcal/kg/day	Route 1 =route 2

Supplemental Table 3

Further information of the included RCTs

Study	Total infection: Events	Total infection: Patients	Infected pancreatic necrosis	Line infection	Bacteremia	Pneumonia	Urinary tract infection	Other type Infection
Abou-Assi, 2002	NJ:5 / TPN:13	NR	NR	NJ:1 / TPN:9	NR	NR	NR	NJ:4(died) / TPN:4(died)
Casas, 2007	NJ:1 / TPN:10	NJ:1 / TPN:5	NJ:0 / TPN:2	NJ:0 / TPN:2	NJ:0 / TPN:3	NR	NJ:1/TPN:0	NJ:0 / TPN:3 (2 SIRS,both infection+ 1 MOF with infection)
Doley, 2009	NJ:29 / TPN:30	NJ:16 / TPN:15	NR	NR	NJ:5 / TPN:8	NR	NR	NJ:24 (FNA(+):5, COS(+):11, drain fluid(+):8) / TPN:22 (FNA(+):11, COS(+):6, drain fluid(+):5)
Du,2015	NJ:1 / NG:0	NJ:1 / NG:0	NR	NR	NR	NJ:1 / NG:0	NR	NR
Eckerwall, 2006	NG:3 / TPN:0	NG:3 / TPN:0	NG:1 / TPN:0	NR	NR	NR	NR	NG:2 (septic) / TPN:0
Entock, 2005	NR	NR	NR	NR	NR	NR	NR	NR
Gupta, 2003	NJ: 1 / TPN:2	NJ:1 / TPN:2	NR	NJ:0 / TPN:1	NR	NJ:0 / TPN:1	NJ:1 / TPN:0	NR
He, 2004	TPN:5 / NNS:8	TPN:5 / Non:8	TPN:5 / NNS:8 (pancreatic infection)	NR	NR	NR	NR	NR
Kalfarentzos, 1997	NJ:6 / TPN:15	NJ:5 / TPN:10	NJ:2 (abscess:1) / TPN:4	NJ:0 / TPN:2	NJ:1 / TPN:3	NJ:2 / TPN:4 (include ARDS)	NJ:1 / TPN:2	NR
Kumar, 2006	NJ:6 / NG:7	NR	NJ:3 (pancreatic aspiration) / NG:3 (pancreatic aspiration)	NR	NJ:2 / NG:3	NJ:1 (tracheal aspiration) / NG:0	NR	NG: 1(bile culture) / NJ:0
McClave, 1997	NJ:4 / TPN:8	NJ:2 / TPN:2	NR	NJ:0 / TPN:2	NJ:1 / TPN:1	NJ:2 / TPN:2	NJ:1 / TPN:2	NJ:0 / TPN:1 (wound infection)
Louie, 2005	NJ:1 / TPN:6	NJ:1 / TPN:4	NJ:1 / TPN:4	NJ:0 / TPN:2	NR	NR	NR	NR
MIMOSA trial	NR	NR	NR	NR	NR	NR	NR	NR
Olah, 2002	NJ:5 / TPN:13	NJ:5 / TPN:13	NR	NR	NR	NR	NR	NR
Petrov, 2006	NJ: 11 / TPN: 27	NJ:7 / TPN:25	NJ:7 (pancreatic abscess:2) / TPN:16 (pancreatic abscess:4)	TPN:5 / NJ:0	NR	NJ:2 / TPN:2	NJ:2 / TPN:4	NR
Powell, 2000	NR	NR	NR	NR	NR	NR	NR	NR
Sax, 1987	TPN:6 / NNS:0	TPN:3 / NNS:0	NR	TPN:3 / NNS:0	TPN:3 / NNS:0	NR	NR	NR
Singh, 2012	NJ:24 / NG:13	Any culture(+): NJ:14, NG:9	NJ:5 / NG:2 (pancreatic aspirate)	NR	NJ:9 / NG:8 (blood culture)	NJ:6 (tracheal aspirate) / NG:3	NR	Bile culture(+) / NJ:4, NG:0
Stimac, 2016	NJ:2 / NNS:0	NJ:2 / NNS:0	NJ:2 / NPO:0	NR	NR	NR	NR	NJ:0 / NNS:0
Wang, 2013	NJ:13 / TPN:24	NJ:13 / TPN:24	NR	NR	NR	NR	NR	Pancreatic sepsis: NJ:13 (incidence% result) / TPN:24
Wu, 2010	NJ:20 / TPN:48	NR	NJ:12 / TPN:39	NJ: 0 / TPN: 1	NR	NR	NR	Wound infection: NJ:8 / TPN:8
Zhang, 2011	NJ:0 / TPN:1	NJ:0 / TPN:1	NJ:0 / TPN:1	NR	NR	NR	NR	NR

ARDS, acute respiratory distress syndrome; COS, culture of operative specimen; FNA, fine needle aspiration; MOF, multiple organ failure; NG, naso-gastric feeding; NJ, naso-jejunal feeding; NNS, no nutritional support; NR, not reported; SIRS, systemic inflammatory response syndrome; TPN, total parenteral nutrition.
