# Supplementary material for: Infection rate among nutritional therapies for acute pancreatitis: A systematic review with network meta-analysis of randomized controlled trials
Source: PLoS One. 2019 Jul 10;14(7):e0219151. doi: 10.1371/journal.pone.0219151 (PMC6620007; doi:10.1371/journal.pone.0219151)
Supplement: S4 Table — (PDF) [file pone.0219151.s006.pdf]

# S4 Table

## Regression by inclusion year and total parenteral nutrition timing

### Regression of inclusion year on overall infection rates

. network meta c, regress(Year) eform(Risk ratio)

Command is: mvmeta _y _S Year , eform(Risk ratio) bscovariance(exch 0.5) longparm suppress(uv mm) vars(_y_B _y_C _y_D)

Note: using method reml

Note: regressing _y_B on Year

Note: regressing _y_C on Year

Note: regressing _y_D on Year

Note: 16 observations on 3 variables

Note: variance-covariance matrix is proportional to .5*I(3)+.5*J(3,3,1)

initial: log likelihood = -41.719253

rescale: log likelihood = -41.491354

rescale eq: log likelihood = -40.643315

Iteration 0: log likelihood = -40.643315

Iteration 1: log likelihood = -40.43699 (not concave)

Iteration 2: log likelihood = -40.427092 (not concave)

Iteration 3: log likelihood = -40.42473

Iteration 4: log likelihood = -40.423982

Iteration 5: log likelihood = -40.42398

Multivariate meta-analysis

Variance-covariance matrix = proportional .5*I(3)+.5*J(3,3,1)

Method = reml Number of dimensions = 3

Restricted log likelihood = -40.42398 Number of observations = 16

| | Risk ratio | Std. Err. | z | P>\|z\| | [95% Conf. Interval] | |
|---|---|---|---|---|---|---|
| _y_B | | | | | | |
| Year | 1.044708 | .0818313 | 0.56 | 0.577 | .8960268 | 1.218061 |
| _cons | 7.09e-39 | 1.11e-36 | -0.56 | 0.575 | 2.3e-172 | 2.22e+95 |
| _y_C | | | | | | |
| Year | 1.018605 | .0401633 | 0.47 | 0.640 | .9428509 | 1.100445 |
| _cons | 5.27e-17 | 4.16e-15 | -0.47 | 0.635 | 3.04e-84 | 9.14e+50 |
| _y_D | | | | | | |
| Year | .7276564 | .1800575 | -1.28 | 0.199 | .4480204 | 1.18183 |
| _cons | 7.3e+276 | 3.6e+279 | 1.28 | 0.199 | 1.3e-146 | . |

Note._y_A, total parenteral nutrition (reference); _y_B, the effect of no nutritional support; _y_C, the effect of naso-jejeunal; _y_D, the effect of naso-gastric.

# S4 Table

## Regression by inclusion year and total parenteral nutrition timing

### Regression of total parenteral nutrition timing on overall infection rates

```
. network meta c, regress(initial) eform(Risk ratio)
Command is: mvmeta _y _S initial    , eform(Risk ratio)    bscovariance(exch 0.5) longparm
suppress(uv mm)    vars(_y_
> B _y_C _y_D)
Note: using method reml
Note: regressing _y_B on initial
Note: regressing _y_C on initial
Note: regressing _y_D on initial
Note: 16 observations on 3 variables
Note: variance-covariance matrix is proportional to .5*I(3)+.5*J(3,3,1)

initial:        log likelihood = -43.420558
rescale:        log likelihood = -42.942637
rescale eq:     log likelihood =  -41.81614
Iteration 0:    log likelihood =  -41.81614
Iteration 1:    log likelihood = -41.653106
Iteration 2:    log likelihood = -41.624167
Iteration 3:    log likelihood = -41.623908
Iteration 4:    log likelihood = -41.623908

Multivariate meta-analysis
Variance-covariance matrix = proportional .5*I(3)+.5*J(3,3,1)
Method = reml                                     Number of dimensions     =      3
Restricted log likelihood = -41.623908            Number of observations   =     16
------------------------------------------------------------------------------
             | Risk ratio   Std. Err.      z    P>|z|     [95% Conf. Interval]
-------------+----------------------------------------------------------------
_y_B         |
     initial |   1.080682   .0746824     1.12   0.262     .9437877    1.237433
       _cons |   .0238332   .0750067    -1.19   0.235     .0000499    11.37728
-------------+----------------------------------------------------------------
_y_C         |
     initial |   1.004745   .0125475     0.38   0.705     .9804506    1.029641
       _cons |   .4333466   .3007194    -1.21   0.228     .1112108    1.688588
-------------+----------------------------------------------------------------
_y_D         |
     initial |   .8772707   .0601114    -1.91   0.056     .7670232    1.003364
       _cons |   175.1116   549.3466     1.65   0.100     .3741028    81966.96
------------------------------------------------------------------------------
```

Note._y_A, total parenteral nutrition (reference); _y_B, the effect of no nutritional support; _y_C, the effect of naso-jejeunal; _y_D, the effect of naso-gastric.
