# Supplementary material for: Infection rate among nutritional therapies for acute pancreatitis: A systematic review with network meta-analysis of randomized controlled trials
Source: PLoS One. 2019 Jul 10;14(7):e0219151. doi: 10.1371/journal.pone.0219151 (PMC6620007; doi:10.1371/journal.pone.0219151)
Supplement: S5 Table — (PDF) [file pone.0219151.s007.pdf]

S5 Table

Summary of SUCRA analysis

Route	Rank Probability				Mean rank	SUCRA
	Rank 1	Rank 2	Rank 3	Rank 4		
**Infected pancreatic necrosis**						
TPN	0.0	0.8	56.2	43.0	3.4	19.3
NNS	1.0	4.4	38.5	56.1	3.5	16.8
NJ	32.8	65.7	1.5	0.0	1.7	77.1
NG	66.2	29.1	3.8	0.9	1.4	86.8
**Bacteremia**						
TPN	0.4	4.7	18.7	76.2	3.7	9.8
NNS	75.6	5.5	8.7	10.2	1.5	82.2
NJ	9.1	45.3	42.3	3.3	2.4	53.4
NG	14.8	44.6	30.3	10.3	2.4	54.7
**Line infection**						
TPN	0.0	41.7	58.3	N/A	2.6	20.9
NNS	11.4	46.9	41.7	N/A	2.3	34.9
NJ	88.6	11.4	0.0	N/A	1.1	94.3
**Pneumonia**						
TPN	5.1	5.8	89.1	N/A	2.7	15.8
NJ	21.3	71.1	7.5	N/A	2.2	41.4
NG	73.6	23.1	3.4	N/A	1.1	92.9
**Other type of infection**						
TPN	14.8	45.9	39.3	N/A	2.2	37.8
NJ	42.5	44.6	12.9	N/A	1.7	64.8
NG	42.7	9.4	47.8	N/A	2.1	47.4

1 NG, naso-gastric; NJ, naso-jejeunal; NNS, no nutrition support TPN, total parenteral nutrition..
